# Open science and ethics in energy research

**DOI:** 10.12688/openreseurope.15707.1

**Published:** 2023-03-31

**Authors:** Raquel Alonso Pedrero, Felipe Van de Sande Araujo

**Affiliations:** 1 Department of Industrial Economics and Technology Management, Norwegian University of Science and Technology, Trondheim, Norway

**Keywords:** open science, research ethics, energy research

## Abstract

Energy research is evolving, with new methodologies, technologies, and challenges, while new communication tools allow for quick and cheap dissemination of information. In contrast, the publication of scientific research is still carried out by specialised journals, which serve a dual purpose as gatekeepers and disseminators but may delay the process of sharing the scientific knowledge. Data used in relevant research is often kept secret, and proprietary code and "black-box" models are barriers to replication. These practices raise ethical concerns as they may hinder the identification of research misconduct. Open science has gained momentum and aims to promote openness, reconnecting with traditional research principles. In this paper, we discuss the ethical and practical implications of the adoption of open science in energy research. Our goal is to give a broad understanding of the benefits and drawbacks of open science and present the ongoing discussions in the research community.

## Plain language summary

Open Science is becoming a key feature in promoting better research practices and reaching citizens and the non-expert public interested in the development of science. In a world where people are becoming more interconnected, academics can now reach a wider part of the population through open-access platforms that bridge the gap with experts. Despite the general acceptance of openness, there is a general lack of understanding of the implications for researchers and all the dimensions involved in fully reaching it (e.g., open methodology, open data).

In this paper, the authors describe what open science is, discuss how it is aligned with traditional research principles and present the benefits and drawbacks for scientists who adopt it. In particular, the authors focus on the domain of energy research, which, differently from other disciplines (e.g., medicine, mathematics), has been lagging in adopting Open Science practices. The paper intends to support research transparency and availability but also present and highlight the difficulties that researchers might encounter before, during and after making their research open to the public.

## I. Introduction

Research has been affected throughout history by the scientific ethos of the moment. For example, in medieval times, the encouraging attitude was to withhold knowledge from the "vulgar multitude", enticing arcane and occult information to a selected group ^1^. While the protection of trade secrets and intellectual rights can be beneficial for entrepreneurship, this behaviour can be detrimental to society if it stands in the way of public access to scientific knowledge, especially regarding matters of common consequence.

Modern societies have gradually incorporated science as a fundamental feature that defines the productive forces, power structures, social classes, or political actions, among other societal features ^2^. Therefore, making science accessible could bring considerable benefits to society and the research community, such as financial savings, increase development of scientific education and dissemination in the Global South, or promotion of sustainable practices ^3^.

In recent years, many disciplines have been prone to adopt open science as common practice, such as astronomy, genomics, or oceanography ^4^. This shift is not so prevalent in energy research, as it is still common to use black-box models and private data that hinder discussion and verification ^5^. Instead, ethical discussion in the energy field has focused on essential questions, such as climate change or energy justice. Considering the influence of the field of research in politics and society and vice versa ^6^, we argue that openness and transparency should be also included as a significant topic for discussion in energy research.

This essay aims to analyse the adoption of open science and evaluate its integration in the energy research domain. In Section 2 we provide a brief overview of the definition of open science and discuss its benefits and drawbacks. Thereafter, we focus on elaborating more in detail on the current application and implications of open science in energy research. Finally, we discuss and conclude with the key elements and lessons learned.

## II. Open science: definition

A widely used definition for open science comes from Michael Nielsen ^7^ and states: "open science is the idea that scientific knowledge of all kinds should be openly shared as early as is practical in the discovery process". A similar and shorter definition can be found in the "Guide to Open Science", where open science is "the idea that scientific research should be openly and immediately shared". Hence, it promotes universal participation and encourages data distribution in standardized formats. This concept of openness in science can be linked to the traditional ethic values stipulated by Robert Merton ^8^ which have been used as fundamental ideals of scientific practice ^9^. The four values are: *communism* (collaboration and cooperation), *universalism* (avoid secrecy and ensure accessibility), *disinterestedness* (relegate individual motives) and *organised skepticism* (to extend previous findings).

Open science goes beyond open data or open access as it provides accessibility to a wider variety of resources such as analysis and methods ^4^. The six principles of open science are open methodology, open source, open data, open access, open peer review and open educational resources.

In open science, the praxis of open methodology is to increase transparency and homogeneity in the documentation of methodological approaches taken in scientific research. This practice is one of the least divisive principles of open science, as good methodological practices have been a top requisite for high-quality scientific publications. The possibility of revising methodological approaches promotes discussions and incentives for the elaboration of new research.

Another principle of open science is the promotion of open source. It is usually framed around the concept of technology or science developed through collective effort and community guidance. Pursuing open source is a rupture from the closed-gates form of development present in some technological platforms. Researchers voluntarily choose to use and develop scientific tools that are available without barriers, allowing for more reproducible experiments and the availability of software.

Adopting open data requires that scientific databases become publicly available. Other researchers are, in this way, incentivised to conduct alternative experiments with the available data. Open data can boost collaboration and promote comparable studies among researchers in line with Mertonian values. Another benefit of openness is that it may reduce the time and resources deployed to gather information. Some studies also examine the potential use of open data as a qualitative metric of scientific success ^4, 10^.

The praxis of open access aims to use the possibilities provided by new communication technologies to disseminate scientific knowledge freely and without discrimination. In some cases, researchers are not required to pay to publish, and everyone can benefit from the electronic distribution of scientific literature.

Quality assurance has been a service provided by the scientific publishing industry. It usually consists of editors making a quick and specific filter and specialists in the subject reviewing papers in more depth. This revision process is often anonymous and voluntary to promote objectivity. Open peer review proposes a similar evaluation process which is conducted in an open and sometimes decentralized environment. This field, however, is still under development, keeping up with the adoption of open access.

Finally, the principle of open educational resources promotes the elaboration of free and accessible materials that can enhance the education of researchers, students, or any person interested in learning science. 

## III. Open science for prevention of bad research practices

In addition to the benefits associated with open dissemination, academia aims to prevent practices that are not considered appropriate when pursuing research through the adoption of open science. We distinguish two types of bad research practices: scientific misconduct and questionable research practices. The former includes behaviours that can be sanctioned according to research ethics regulations, whereas the latter, despite being considered not desirable, is not associated with penalisation procedures. The U.S. Office of Research Integrity defines scientific misconduct as:

*“[...] fabrication, falsification, or plagiarism in proposing, performing, or reviewing research, or in reporting research results.*

*(a) Fabrication is making up data or results and recording or reporting them.*

*(b) Falsification is manipulating research materials, equipment, or processes, or changing or omitting data or results such that the research is not accurately represented in the research record.*

*(c) Plagiarism is the appropriation of another person's ideas, processes, results, or words without giving appropriate credit.*

*(d) Research misconduct does not include honest error or differences of opinion”*

Behaviours that belong to the category of questionable research practices are, for example, selecting specific references to back up hypotheses, not reporting unexpected results, or not stating all the investigation conditions. Given the greater likelihood of researchers committing these infringements, some scientists claim that these are the real source of harm to research integrity ^11, 12^.

Besides the possibility of individual punishment for scientific misconducts, all these bad research practices have long-term consequences for the whole scientific community such as mistrust in science, loss of social justification, and the generation of misguiding insights that risk the role of science in society.

Open science can prevent these undesirable research practices by inducing the reproducibility of research. Allowing other researchers to reuse the data and methods (e.g., code) used for the publications enhances the chance to detect bad research practices.

*A. Barriers and shortcomings of open science*

The three main drawbacks of sharing scientific resources are, according to Feigenbaum and Levy ^13^, additional effort, interference with future work, and verification as a double-edged sword.

It is not unusual to find researchers who consider that preparing resources for external interested parties is time-consuming or negative for their future research. In an experiment two hundred researchers were asked to share the supplementary material as promised in their articles ^14^. Only 40% shared the requested material, and most of the rejections claimed that it was not worth their time. A similar study received similar refusals arguing that sharing their resources could interfere with their ongoing projects ^15^.

Enhancing verification and reviewing processes could be considered a double-edged sword because it could improve the quality of research ^16^, but also ease the identification of non-voluntary questionable practices that may harm researchers’ recognition. The possibility of being involved in professional controversies may increase fear toward the adoption of open science ^13^, especially in a competitive environment. In this regard, competition in the research community has proven to limit collaboration and sharing practices ^17^ or has been even considered as the root of bad research practices ^18^. While professional interests might be seen as a questionable reason for not engaging in open science, other reasons can be aligned with ethical concerns such as patient/customer privacy, confidentiality, and copyright issues.

Another barrier to implementing open access is related to the publishing industry revenue structure ^19^. Mainstream journals perform several functions in the current research publication environment, such as archival research information for posterity, registration of scientific discovery to establish precedence, dissemination of scientific knowledge, and certification asserting rigour and significance of contributions ^20^. Decoupling those functions into different services could promote a different stream of revenue for journals, for instance, authors could pay to register a discovery, or pay for a review from an editor, and in exchange, the journal could allow open access and archival function with no charge to the readers.

Understanding open science as a synonym of quality could lead to negative outcomes as it does not warrant appropriate methodologies or well-oriented questions. Open science could lead to “flaw studies or junk science” if researchers stick their studies to available resources instead of pursuing valuable investigation paths ^10^.

Open science also lacks maturity in the tools that warrant a minimal level of quality, which is attained through the editorial process in most journals nowadays. Some open access repositories, especially preprint servers, do not perform any kind of pre-publication analysis. Although post-publication criticism is possible, it is not always visible or arranged in a relevant manner. A large number of publications without any form of analysis could paradoxically foster more fabrication, falsification, and plagiarism. The benefit of transparency is then lost under unrestricted publication.

Quality mechanisms for open science are being developed, and open peer review is already under development. Other means, such as citations, are already decentralized and can be maintained. Some studies ^7, 21^ identify the quality metrics currently used, e.g., citations and impact factor, as tools that do not promote the correct incentive. Because of this, alternative metrics have been suggested, and they are called collectively “altmetrics” ^22, 23^, some of which are already being adopted by the traditional publishing industry.

## IV. Open science and energy research

A characteristic of energy research is its repercussions on political decision-making and, consequently, on society and the environment ^24^. Not following the scientific ethos could increase mistrust, not only in science but also in governments and legal administrations. Transparency and accessible energy science are as important as ever with global concerns such as climate change, supply crisis caused by war or the economic recovery after the coronavirus disease (COVID-19) pandemic.

Open science has multiple positive outcomes (e.g., collaboration, identifying bad practices). However, Pfenninger *et al.* ^25^ identify four prevalent outcomes that fit with energy research practices. These are: (1) easing the identification of errors in models and assumptions, (2) improvements in the researchers' productivity, (3) incorporating more scientific arguments in societal debates, and (4) the promotion of better cooperation between policy and research.

Another positive aspect of open science is its ability to homogenise data and metadata. Ensuring a common understanding is particularly important in fields that are associated with multiple disciplines as it is the case of energy research (e.g., operations research, macroeconomics, power system engineering, energy policy). With such a variety of understandings about the same topic, one usually encounters different terminologies, assumptions, or modelling techniques ^4^. This heterogeneity may hinder the exchange of insights between disciplines, resulting in studies with a narrow scope and disciplinarily myopia ^26^, a problem that could be solved by enhancing collaboration.

In energy domains where innovation is patentable, private capital might invest in research and development for commercial purposes. In this setting, it is expected that the societal cost of not having access to information is not as significant as the cost of burdening the risk of failure. This applies particularly well to research oriented towards developing business models and new technologies (e.g., new battery materials, designs of wind turbines, software for operating energy management systems). Other domains related to energy science rely mostly on public funding, such as energy policies or environmental studies. It is a matter of debate whether the knowledge developed with public funding should have restricted publications and data and use software that charge a fee to grant access, thus being less reproducible.

A recent paper by Hart *et al.* ^21^ lists many benefits of open science to engineering, but many of the impacts can be extrapolated for research in similar fields, such as energy. The authors point out that filing for patents is often more costly than whatever revenue is obtained, as it is a time-consuming process which take research away from its focus, and it is not necessary for obtaining private funding. The authors conclude with an analogy that will be familiar to many using optimisation tools: open science has a similar effect on research as a less constrained model, usually improving the objective function.

Friesike *et al.* ^7^ make a parallel between open science and open innovation, identifying many convergent aspects and suggesting that the adoption of open science might describe an inevitable paradigm shift. They present several trends that shape the future of open science, which are a higher acceptance of open access, the emergence of open reviewing and new quality measurements, new distribution models for collective knowledge creation, more interdisciplinary research, outsourcing of research from within small and medium companies and intellectual property trading. Those trends can be identified within the energy field, especially regarding research collaboration between companies and universities.

To promote open science in the field of energy research, Howells *et al.* ^27^ propose a guideline on how energy modellers could present their research to experts and non-experts. Key recommendations are compactness to ease the learning process, deployment of free-access programming languages (e.g., Python), explanations in plain English, and presentation of all data input. Similarly, Hilpert *et al.* ^28^ describe an approach for open modelling energy systems to tackle more complex and wide-ranging problems, for which collaboration can provide the best answer.

In recent years, there has been a growing interest in embracing open science in the energy field, particularly from academia and political organisations. The OpenMod (Open Energy Modelling) initiative arose in 2012 due to the concerns around the deployment of "black-boxes" in the energy domain that could undermine the reputation of the field and result in inefficiencies for the researchers. This international initiative has united open access resources ranging from data, models, and references to other open science initiatives related to the energy field.

**Figure 1.**
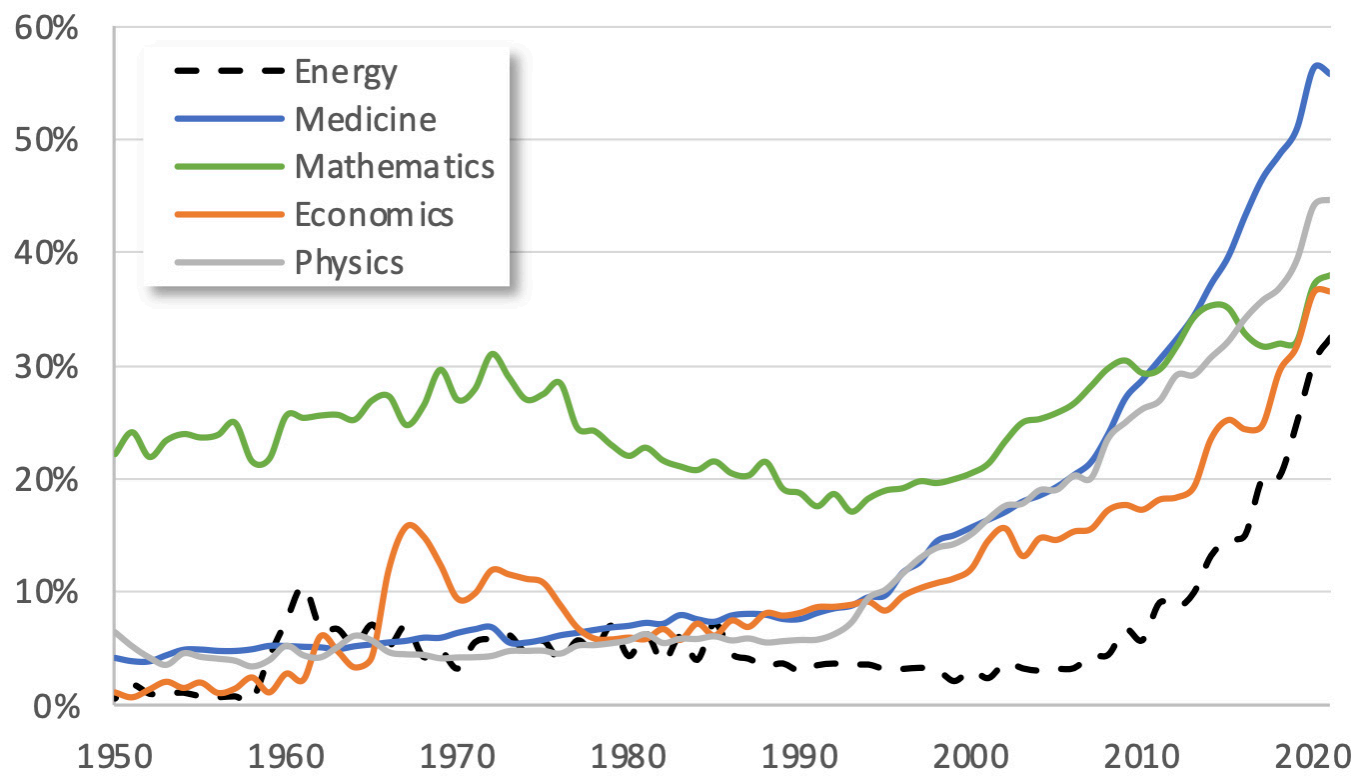
Development of the percentage of open access over all publications for different research fields. Data retrieved from Scopus.

Another example of an effort to incorporate open science in energy research is the Horizon2020 framework. One of its goals focuses on bringing science closer to society by promoting the three O's as strategies: open innovation, open science, and open to the world. The EU Open Data Portal, following the ethical outlines, contains free-access datasets of diverse research domains. The number of entries for the energy category is remarkably low compared to other research fields (1,094 datasets in "Energy" compared to 4,033 in "Population and Society", 3,093 in "Environment" or 2,952 in "Science and Technology"). These numbers can have their roots in the number of projects funded, but they might also reveal that there are substantial barriers to open science in the energy domain. When analysing the percentage of open access publications, we find that energy research falls behind domains such as medicine or mathematics (see Figure 1).

 Many energy firms and research institutes largely rely on the income provided by owning energy data and models (e.g., utilities, consultants). Due to the crucial role of models in their economic performance, open science faces a strong barrier within these organisations.

New communication technologies can pose a positive and necessary change in the energy landscape. Recently, new movements promoted the unrestricted use of collaborative tools (e.g., OpenMod, European Data Portal, OpenEI), bridging the current energy research environment and the Mertonian ideals based on open science. Also, high-profile scientific journals already offer some degree of open-access space for publication (e.g., Energies, Energy and Policy Research).

Not pursuing openness when presenting energy models could lead to fewer repercussions in the scientific field. This is a problem that leads to more discrepancies between decision-makers and energy researchers ^26^. Lawmakers tend to criticise the possibility of energy models being the product of energy lobbies. Therefore, transparency is required when presenting assumptions, inaccuracies, and limitations of models to ease the understanding of insights from science and increase credibility. Openness can be considered a vital practice for energy research as it has considerable implications in its applicability.

Moreover, these tools are usually developed over the years. Thus, their complexity may be an obstacle to new users. As a result, an interagency review is deployed, for example, in the U.S. to check the different results and evaluate the models' reliability ^26^. In this way, decision-makers do not need to deeply comprehend the models to understand their insights, assumptions, and limitations. Although openness at this level overcomes the accessibility barrier for policymakers, it does not guarantee appropriate disclosure of the results to the rest of the population, resulting in a society lacking the knowledge to understand the reasons for energy policies.

As in other scientific fields, energy research is difficult to comprehend without some education. Therefore, energy-related knowledge should be taught and disseminated through open educational resources that reach a higher share of society. Universal access to education is becoming more common in the energy domain with conferences and workshops open to the general public (A.C.M. e-Energy, D-A-CH+ Conference on Energy Informatics or Future Technologies Conference) and Open Courseware uploaded on platforms like YouTube or Coursera.

## V. Discussion

Open science is a multidimensional practice that involves more than sharing of data and can be directly associated with traditional research ethics. It can help to lower the likelihood of research misconducts, disseminate knowledge worldwide, ease collaboration among researchers, industries, and decision-makers, and could enhance the quality of research, either by reducing rework or by redirecting new research.

Interestingly, the practices within open science enhance each other. For example, open education is an enabler of the use of open access resources, as it gives the reader more knowledge to interpret results and methodology innovation. A second example is how the effectiveness of open peer-review can be enhanced by open data and open methodology.

Despite its benefits, the adoption of open science is not enough to avoid scientific ailments. Active participation from the readers is required to ensure discussion and validation of the published resources, as allowing for universal access is, in itself, not a measure of quality. The same applies to scientific misconduct because shared resources need to be analysed to identify flaws. Open science requires engagement from researchers or content creators as well as from the recipients of the resource.

Nevertheless, ensuring engagement seems to be a major obstacle to the adoption of open science. Researchers may look at it as a time-consuming activity or perceive it as having a negative impact by interfering with the prospect of future projects. The negative view towards open science is enhanced within highly competitive sectors, in which researchers’ time and proprietary resources are key to getting founded and gaining prestige. Another major obstacle is related to the structural changes required on the publishing side, including new quality metrics and open peer-review.

As researchers in the energy field, we see how it can benefit from open science principles. Based on our study, the most prevalent benefits are improving collaboration and linkage between multiple disciplines within the field, sharing standardized data and methodologies, and enhancing communication with decision-makers by allowing more access to information and education.

It is visible that the adoption of open science in energy research is progressing, as it is reflected in open access being a requisite for funding, in the increase and development of projects fully oriented to open science (e.g., Open Entrance), and in the prevalence of workshops and conferences with public access.

Despite the progress toward openness, research in the energy field faces significant barriers. Energy-related industries are more likely to fund private projects or limit the publication of data and findings, which could threaten their prestige, credibility, or market position. Moreover, data privacy (e.g., end user consumption patterns) and energy security concerns are legitimate barriers that might be slowing down the integration of open science. Those limitations and shortcomings do not constitute reasons not to adopt open science but cautionary measures to be considered when choosing it.

We agree that open science is undergoing a paradigm shift, one that should be undertaken while considering its benefits and drawbacks. The purpose of this paper is to update this discussion and keep it at the forefront, assuring that better collaboration and research practices will help us all in the energy field to tackle the great challenges ahead.

## Ethics and consent

Ethical approval and consent were not required.

## Data Availability

No data are associated with this article.
