# Open science in energy research

**DOI:** 10.12688/openreseurope.15707.2

**Published:** 2025-01-22

**Authors:** Raquel Alonso Pedrero, Felipe Van de Sande Araujo

**Affiliations:** 1 Department of Industrial Economics and Technology Management, Norwegian University of Science and Technology, Trondheim, Norway

**Keywords:** open science, research ethics, energy research

## Abstract

Energy research is evolving, with new methodologies, technologies, and challenges, while new communication tools allow for quick and cheap dissemination of information. In contrast, data used in relevant research is often kept secret, and proprietary code and non-transparent models are barriers to replication. Also, scientific research is still published in subscription-based journals, hindering knowledge sharing. These practices raise ethical concerns not only stop the dissemination of research but also hinder the identification of research misconduct. Open science has gained momentum and aims to promote openness, reconnecting with traditional research principles. In this paper, we discuss the implications of adopting open science in energy research, examine its benefits but also drawbacks, and present the ongoing discussions in the research community.

## Plain language summary

Open science is key to promoting better research practices and reaching citizens and the non-expert public interested in the development of science. In a world where people are becoming more interconnected, academics can now reach a wider part of the population through open-access platforms. Despite the general acceptance of openness, there is a general lack of understanding of the implications for researchers and all the dimensions involved in fully reaching it.

In this paper, the authors describe open science and present the benefits and drawbacks for scientists who adopt it. In particular, the authors focus on its application in energy research and its particularities in adopting open science practices. The paper intends to support research transparency and availability but also present and highlight the difficulties researchers might encounter before, during and after making their research open to the public.

## I. Introduction

Research has been affected throughout history by the scientific ethos of the moment. For example, in medieval times, the encouraging attitude was to withhold knowledge from the "vulgar multitude", enticing arcane and occult information to a selected group ^1^. While the protection of trade secrets and intellectual rights can be beneficial for entrepreneurship, this behaviour can be detrimental to society if it stands in the way of public access to scientific knowledge, especially regarding matters of common consequence. In 1942, Robert Merton introduced four ethical values that would constitute the ethos of modern science and scientific practice ^2^. The four values proposed by Merton are *communism* (public ownership of knowledge), *universalism* (everyone can do science), *disinterestedness* (relegate individual motives) and *organised skepticism* (organised assessment of scientific contribution). Although these values have received criticism for being excessively idealistic ^3^, the research ethos has aligned with them and has progressed towards openness and transparency.

Modern societies have gradually incorporated science as a fundamental feature that defines the productive forces, power structures, social classes, and political actions, among other societal features ^4^. Making science accessible brings considerable benefits to society and the research community, such as financial savings, increased development of scientific education and dissemination in the Global South, and promotion of sustainable practices ^5^. As a result, examining and promoting how science should be communicated and shared are concerns addressed by modern initiatives like open science.

Many disciplines, such as astronomy, genomics, or oceanography, have been prone to adopt open science as common practice in recent years ^6^. This shift may not be so prevalent in energy research, where it is still common to use black-box models ^1^ and private data that hinder discussion and verification ^7^. While the energy field has focused on ethical questions related to climate change and energy justice, it may not have fully embraced open science practices.

Given the significant influence of energy research on politics and society and vice versa ^8^, we argue integrating open science principles can enhance research integrity by promoting transparency, reproducibility, and accountability. This essay analyses the adoption of open science and evaluates its integration in the energy research domain. We do so by exploring recent trends of adopting open science in energy research (e.g., research projects, publications, initiatives) and recent literature on the topic. Also, we intend to look at the positive impact and challenges of the research ethos of openness.

The paper starts with an overview of open science's general definition, implementation, and benefits in Section 2. Section 3 discusses its drawbacks, while Section 4 elaborates on open science's current application and implications of open science in energy research. Finally, we discuss and conclude with the key elements and lessons learned.

## II. Open science: definition

Multiple definitions for open science have been proposed in the last decades, and its interpretation has evolved ^9^. Today, one of the most used definitions is the one proposed by Michael Nielsen ^10^, which describes open science as: "the idea that scientific knowledge of all kinds should be openly shared as early as is practical in the discovery process". This definition emphasises two core values: universal participation, related to openness, and data and knowledge distribution at all stages of science.

### A. Principles of open science

Today, concepts like *open source* are well-established among researchers and academics. Open-source platforms embody the concept of universal participation by providing accessible, free, and transparent software developed and shared by scientists. However, openness should extend across various stages of scientific practice. As such, open science encompasses seven core principles ^11, 12^:

1. **Open methodology** increases transparency and homogeneity in the documentation of methods. The possibility of revising methodological approaches promotes discussions and incentives for the elaboration of new research. As such, it is one of the least divisive principles of open science, as good methodological practices are top requisite for high-quality scientific publications.
2. **Open data** can boost collaboration and promote comparable studies among researchers. It allows different researchers to conduct alternative experiments with the available data and reduces the time and resources deployed to gather information. Some studies claim that the reproducibility of research through open data should be considered a qualitative metric of scientific success, given the benefits it brings to the scientific community ^6, 13^. Moreover, multiple studies demonstrate that papers attached to open data receive more citations than their counterparts ^14, 15^. Adopting open data requires that scientific databases become publicly available.
3. **Open peer review** proposes an evaluation process conducted in an open and sometimes decentralised environment. Traditionally, quality assurance has been provided by the scientific publishing industry, where editors make a first filter and then specialists in the subject review the content in more depth. This revision process is often anonymous and voluntary to promote objectivity. Open peer review aims to involve the entire community in the review process, eliminate the anonymity of the reviewers and authors, and ensure traceability throughout the review.
4. **Open hardware** entails the distribution of physical objects to share knowledge of their design and function. The possibility of reusing and sharing hardware limits the necessity of duplicating physical objects, contributing to environmental sustainability. It also promotes innovation by allowing scientists to improve existing hardware using a community-driven approach to hardware development.
5. **Open source** promotes the dissemination of research software like code. Research software is defined as “software that is used to generate, process or analyse results that you intend to appear in a publication” ^16^. This encompasses various tools, including source code, binaries and web services. It spans from short, ad hoc scripts created by researchers to software rigorously developed for a mission-critical process ^17^. This approach supports transparency, as users can inspect the code for security and functionality. The development of open-source software might encourage innovation and advancement of knowledge through researchers’ collaboration.
6. **Open access** aims to disseminate scientific publications freely and without discrimination. In some cases, researchers are not required to pay to publish, and everyone can benefit from the electronic distribution of scientific literature. . There are two main paths to open access: the Green Open Access, where free access to the published work is delayed, and the Gold Open Access, where the work is made immediately open upon publication by the publisher.
7. **Open educational resources** promote the creation of free and accessible materials that can enhance the education of researchers, students, or anyone interested in learning science.

### B. Prevention of scientific misconduct and questionable research practices

Beyond the benefits of openness at all stages of research, open science aims to prevent practices considered inappropriate by the research community. We distinguish between two inappropriate practices: scientific misconduct and questionable research practices.

Scientific misconduct includes behaviours and activities that can be sanctioned according to research ethics regulations. The U.S. Office of Research Integrity defines scientific misconduct as *“[...] a fabrication, falsification, or plagiarism in proposing, performing, or reviewing research, or in reporting research results. […] Research misconduct does not include honest error or differences of opinion”*

Meanwhile, questionable research practices are activities that, despite being considered undesirable, are not associated with penalization procedures. Some examples of questionable research practices are selecting specific references to back up hypotheses, not reporting unexpected results, or not stating all the investigation conditions. Given the greater likelihood of researchers committing these infringements, some scientists claim that these practices are the real source of harm to research integrity ^18, 19^.

All these inappropriate research practices have long-term consequences for the whole scientific community, such as mistrust in science, loss of social justification, and the generation of misguiding insights that risk the role of science in society. Open science can prevent these undesirable research practices by inducing transparency, debate and reproducibility of research.

### C. Applying open science

With open science, the research practices by inducing the reproducibility of research. Allowing other researchers ecosystem transitions from a centralised approach, where resources are only available to reuse the some, to a decentralised system offering software, data, methodologies, and methods knowledge to many. However, making resources available leads to other questions, such as: Where can I find adequate resources? Which tools are needed to find what I am looking for effectively? How do I deal with different types of digital resources (e.g., code) used for data types)? In which degree I am allowed to use this data?

In an attempt to answer some of these questions and enhance the practices of researchers, Wilkinson *et al.* ^20^ propose the publications enhancesFAIR principles to guide stakeholders on how to manage and steward their research data effectively. These principles state that all data resources are:

1. Findable: Data should be easy to locate with well-described metadata (i.e., unique identifiers, identification of metadata, registered in searchable resources)
2. Accessible: Data and metadata should be retrievable through standard, open protocols. In particular, metadata must consistently be accessible, even in the chance to detect badabsence of data, in order to comprehend its deployment context.
3. Interoperable: Data should be structured using formal and understandable language, allowing integration with other data resources. Using consistent terminology and references to other data resources to support data interoperability is recommended – see Open Ontology later in this paper.
4. Reusable: Data must be comprehensively documented (e.g., the usage of a license, the name of creators) and should adhere to community standards to ensure its utility in future research.

Lamprecht *et al.* ^17^ extended these principles to research software, addressing the complex dependencies and dynamic nature of software to research data. For instance, the authors emphasised the importance of version control, documentation, and community practices in ensuring that software remains FAIR. Although the development of the FAIR principles was not explicitly framed as an attempt to promote Open Science, they are undeniably driven by the same pursuit of openness, transparency, and collaboration. More explicitly, they aim to facilitate the development of the pillars of open data and open-source software.

Another source supporting researchers and stakeholders on how to adhere to Open Science is “The Open Science Training Handbook” ^21^. This document includes definitions, examples, and practices for delivering Open Science principles. Also, Science Europe, the European Research Council and the European Commission founded the Plan S and the alliance cOAlition S to help publishers implement the principles of open access in their business models.

## III. Barriers and shortcomings of open science

Despite the benefits of open science in disseminating knowledge and preventing inappropriate practices, it also faces barriers and may present shortcomings.

### A. Additional effort and time consumption

One significant barrier is the additional effort required to implement open science ^22^. Many researchers view the preparation of resources for external parties as time-consuming and detrimental to their future research. For instance, in one experiment, only 40% of 200 researchers shared supplementary materials as promised, with most refusals citing the effort required as not worthwhile ^23^. In addressing this concern, “The Open Science Training Handbook” ^21^ recommends disseminating resources without processing them. The authors assert that if the original resources contributed to scientific outcomes, then they are deemed sufficiently credible for publication.

### B. Impact on future work and competitiveness

Two other barriers to implementing open science are the impacts on future work and competitiveness ^22^. One study found researchers reluctant to share their resources, fearing it would interfere with the publication of ongoing projects ^24^. Researchers may prefer to keep their resources private if they believe sharing them could jeopardise their future publishing and funding opportunities. However, recent evidence demonstrates that articles sharing their data via public repositories have a higher citation impact ^15^, which is a typical measure of competitiveness and research value.

Researchers may hesitate to adopt open science practices because it could harm their recognition and competitiveness ^22^. Worries about competitiveness have been proven to reduce the willingness of researchers to engage in collaborative and sharing practices ^25^ and might contribute to the emergence of inappropriate research practices ^26^.

Another concern on open science and competitiveness is the balance between authorship protection and openness. The idea is that protecting authors through intellectual property (IP) produces more innovation and benefits for society as authors feel secure about being attributed their contributions. To ensure that authors make their work openly accessible, the European Union ^27^ suggests the creation of an Office for Free Intellectual Property Rights and Open Science to ensure that both dissemination paths have the same support and recognition.

### C. Ethical and legal concerns

Ethical concerns may further complicate the implementation of open science. Fundamental ethical principles on privacy mandate the protection of patient/customer privacy and confidentiality. In some research domains, pursuing open data may bring concerns about leaking sensitive information, and, in some cases, it may be illegal ^28^. As previously indicated, IP rights represent a significant obstacle to implementing open science ^27^. From a legal standpoint, researchers who utilise proprietary software protected by copyrights are legally prohibited from sharing the software publicly. This prohibition impedes the utilisation of open software, hardware, and data, limiting researchers from publishing their complete workflow.

### D. Publishing industry challenges

Preprint servers and new forms of research dissemination are challenging the publishing industry in their gate-keeping role. Still, the critical task of research evaluation remains largely in the peer-review process offered by traditional journals ^29^. Mainstream journals perform several functions, such as archiving research, registering scientific discoveries to establish precedence, disseminating scientific knowledge, and certifying the rigour and significance of contributions. Decoupling these functions could allow smaller and independent players to compete for those services, empowering researchers to choose what fits them best. A decoupled publishing industry where mainstream journals cease to be an “all-in-one" solution may offer less resistance to adopting Open Peer Review ^30^.

### E. Quality concerns

 Open resources are not necessarily a synonym of quality. Open resources are not guaranteed to apply appropriate methodologies or well-oriented research questions, especially if they have been published without post-publication reviews. Open science might result in “flawed studies or junk science” if researchers limit their studies to available resources instead of pursuing valuable investigation paths ^13^. Traditionally, the editorial process in academic journals is responsible for guaranteeing a level of quality. Now, many open-access repositories, especially preprint servers, do not perform pre-publication analysis on the quality of the submissions. Although post-publication criticism is possible, it is not always visible or arranged in a relevant manner. This could foster more fabrication, falsification, and plagiarism. The benefit of transparency is then lost under unrestricted publication. This ceases to be a problem with the general adoption of open peer review (e.g., Open Research Europe), which could be extended to preprints as well. Meanwhile, preprint servers could offer a “health warning”, clearly informing the readers that the published material is not peer-reviewed.

Furthermore, the concept of quality is conventionally associated with metrics such as citations and impact factors, which have been recognised as inadequate instruments for promoting quality research ^31^. Impact factors have been described as measurements that are " *rather slow, closed, and biased by social group effects*" ^9^. In contrast, citations under open science are managed and maintained across various platforms rather than in traditional centralised systems. This decentralisation facilitates the use of alternative metrics (altmetrics) to account for a broader range of factors ^21^ and provide a more comprehensive perspective of research impact and quality. Altmetrics can be aggregated using tools that, for example, reveal the number of "mentions of a data set in blog posts, tweets and mainstream media".

## IV. Open science and energy research

This section connects the concepts related to open science, discussed until now, with practices in energy research. Energy research is understood as a multidisciplinary scientific domain focused on applications in the energy sector. The disciplines forming energy research include domains such as politics, ethics and sociology, as well as physics, mathematics and economics.

Given its applied and broad nature, energy research has notable repercussions on political decision-making and, consequently, on society and the environment ^32^. Adhering to the scientific ethos of openness, which ensures transparency and reproducibility, is essential to maintaining public trust in science, governments and legal administrations. Transparency and accessible energy science are as important as ever with global concerns such as climate change, supply crises caused by war or the economic recovery after the coronavirus disease (COVID-19) pandemic. Transparency should encourage evidence-based policymaking while discouraging the fabrication of evidence for political purposes ^33^.

A notable example of mistrust stemming from a lack of transparent and accessible energy research is the concern that lobbies or interest groups could influence its results. In quantitative research, where the tools used are mostly mathematical models, results are particularly sensitive to the data selection, allowing for potential pressure from interest groups to sway the interpretation and generation of results. To mitigate this mistrust, it is crucial to prioritise openness. This can be achieved by implementing open methodology (clear documentation of the methods), open source (providing access to the tools used), and open data (making data accessible). Such transparency is key to identifying assumptions, inaccuracies, and limitations in research and essential for recognising and addressing potential pressures from interested parties. 

### A. Benefits of open science in energy research

Transparency on models and assumptions not only addresses mistrust from society but also benefits the development of the research domain by enhancing feedback and enabling the correction of errors. Pfenninger *et al.* ^34^ identify three other beneficial outcomes of adhering to open science in energy research: increased researchers’ productivity, scientific arguments in social debates, and cooperation between policymakers and energy researchers. These three outcomes align with the motivations proposed one year later by Morrison ^33^ for making energy system modelling open. These are public transparency, scientific reproducibility and open development.

The outcomes proposed by these authors can be linked to specific elements of open science. Open source and open data can be identified as key principles contributing to researchers’ productivity. Software and data are particularly prone to reusability, which helps avoid double work. For example, in the authors’ experience as energy researchers, acquiring anonymised electricity consumption from past projects has saved many hours and resources. Some researchers avoid sharing sensitive data because of data protection issues. Yet, it is possible to emphasise anonymisation and privacy when handling data, enabling sharing even for data deemed sensitive This situation demonstrates that promoting open data is feasible if effective data management protocols are prioritised in energy research projects, aligning with the growing trend of open access initiatives in Europe.Making knowledge accessible through open access increases dissemination and, consequently, the presence of scientific arguments in social debates and cooperation between policy and research. However, while open access makes academic articles in the energy domain available to the public, they are typically written with jargon and in a style that may not be accessible to non-experts. This can hinder the direct impact of such knowledge on social debates or policymakers unless researchers actively engage in translating and communicating their findings to these audiences. Although one can argue that open access does not directly translate into these benefits, it promotes accessibility to researchers’ work. This can foster richer public discussions among experts, allowing for the exchange of scientific arguments. Such interactions can provide a deeper and more diverse pool of scientific insights.

Another positive aspect of open science is its ability to homogenise data and metadata ^35^. An effort to create an open energy ontology that can build a common conceptualisation of the energy domain can be seen in Boosherhi *et al.* ^36^. Modelers focused on energy use cases may find it difficult and costly to use semantic models with existing ontologies ^37^, which leads to the need for a centralised repository, the development of more use cases, and better harmonisation and standardisation in collaboration with industry. A rich analysis of the importance of availability and homogeneity of metadata shows that accessible, standard and machine-readable metadata is essential for further development of research in the field, which can be facilitated by the increased digitalisation within the sector ^35^. The authors point out that this is not currently the case, and energy data (and metadata) is segregated into islets, each with its own standards and nomenclature. One of the underlying factors for data segregation may be the heterogeneity of disciplines within energy research (e.g., operations research, macroeconomics, power system engineering, and energy policy). With such a variety of disciplines, one usually encounters different terminologies, assumptions, or modelling techniques ^6^ that may hinder the exchange of insights and result in studies with a narrow scope and disciplinarily myopia ^38^.

The implementation of open data platforms that are accessible to all energy researchers could significantly alleviate these challenges by providing a common repository while also promoting consistency in the structuring and sharing of data and metadata. By adhering to standards, such platforms can help to harmonise diverse terminologies and, thereby, facilitate collaboration and data exchange. Furthermore, they may minimise redundant efforts.

### B. Barriers and limitations of open science in energy research

In the energy field, open science faces barriers tied to competition and legal concerns. Many research institutes economically depend on owning energy data and models to secure funding and offer consultancy services. Because of this, researchers often see openness as a threat to their economic performance and competitiveness. They fear that making their work open will allow competitors to replicate it. However, one can argue that these tools are often too complex to replicate easily and, more importantly, that if so, replicability is key to scientific progress. To address these concerns, energy researchers use legal tools like property rights (e.g. MIT License) to protect their work and manage its distribution and use.

Still, the complexity of energy research models may hinder the ability of non-experts to obtain the key information from the outcomes. Researchers adhering to open science still cannot guarantee that new users appropriately interpret their outcomes. Clear documentation and descriptions of the assumptions made, as proposed in the FAIR principles, are needed. This is particularly relevant for energy policymakers who rely on outcomes from complex energy models designed based on someone else’s assumptions. To facilitate the understanding of assumptions in energy models, Munson ^38^ proposes interagency reviews to openly share the assumptions of all relevant models in the U.S. energy policy.

Clear documentation and descriptions may also facilitate the discussion between expert fields. However, as pointed out by Morrison ^33^ one potential disadvantage of this collaboration is the overload of work to researchers and developers from external contributions to the code/software that could overweight the benefits.

Furthermore, energy research can be closely tied to innovation, as it is common for research to be translated into commercial applications. When innovation is patentable, private investors might fund research to turn ideas into profits, being willing to take on the risk of failure. In those cases, information from research may not be immediately accessible to the public due to proprietary restrictions, and thus more unlikely to adhere to open science. However, the societal cost of limited access to information may not be as significant as the cost of bearing the risk of failure. This applies particularly well to research oriented towards business models and new technologies (e.g., battery materials, wind turbine design, energy management systems).

Conversely, other domains within energy research rely mostly on public funding. Some examples are energy policy and environmental studies. In these cases, it is a matter of debate if knowledge developed from public funding should be disseminated through restricted publications and if data and software adhered to fees. Plan S is envisioned as an initiative for open-access publishing, and starting in 2018, it has achieved big steps in the open-access publication of results from research funded by public grants.

Another area that faces challenges adhering to open science is research on critical infrastructure and cybersecurity. These fields are essential for securing key societal technologies, such as electricity and gas networks. As with the trade-off between innovation and restricted access to information, the risk of exposing sensitive security data and methods outweighs the benefits of making it public. In this case, the potential harm might be too great to justify complete openness.

### C. Integration of open science in energy research

In the last years, multiple initiatives have focused on increasing the adoption of open science within the energy scientific community. The OpenMod (Open Energy Modelling) initiative was launched in 2012 out of a concern that using "black boxes" in energy research might harm the field's reputation. This international initiative has united open science resources (e.g., data, models, and references) and connected them to other open science projects in the energy sector. The goal is to make the field more transparent and trustworthy.

Another example of an effort to incorporate open science in energy research is the Horizon2020 framework and its requirements to adhere to openness. One of its goals focuses on bringing science closer to society by promoting the three O's as strategies: open innovation, open science, and open to the world. The EU Open Data Portal, following the ethical outlines, contains free-access datasets of diverse research domains. Recently, new movements promoted the unrestricted use of collaborative tools (e.g., OpenMod, European Data Portal, OpenEI), bridging the current energy research environment and the open scientific ethos. Also, high-profile scientific journals already offer some open-access space for publication (e.g., Energy and Policy Research).

To promote the integration of open science in energy modelling, researchers have developed various frameworks, guidelines and tools to enhance transparency, accessibility and collaboration. Howells *et al.* ^39^ propose a guideline for presenting research effectively to experts and non-experts, emphasising compactness to simplify the learning process, using open source programming languages such as Python, explanations in plain English, and clear presentation of all data inputs to support reproducibility. Pfenninger *et al.* ^40^ compiled strategies to increase the transparency of “black-box” models, advocating for open-source practices to promote reproducibility and improve trust in modelling efforts. Similarly, Huebner *et al.* ^41^ introduced the “TReQ” approach, a set of tools aimed at reducing questionable research practices. These tools include preregistering analysis plans, adhering to reporting guidelines, sharing preprints, and making data and code openly available, addressing different stages of the research process and mitigating issues like data manipulation, insufficient details of methods, and lack of reproducibility. Complementing these efforts, Cao *et al.* ^42^ provide a checklist for transparency in energy scenario studies, highlighting the importance of clearly communicating assumptions. Efforts to foster trust and collaboration between stakeholders have also been explored, such as the “Open and Reproducible Use Cases for Energy” (ORUCE) methodology proposed by Hodencq *et al.* ^43^, which outlines a transferable workflow for applying open energy modelling principles while minimising errors and biases. New online resources such as “renewables.ninja” and “ourworldindata.org” are open data platforms that facilitate access to data for energy researchers.

In recent years, there has been a growing trend among energy modellers to embrace open-source approaches by creating reusable and extendable energy models. For instance, Hilpert *et al.* ^44^ developed the Open Energy Modelling Framework designed to facilitate collaborative and transparent energy system modeling. Similarly, EnergyModelsX was introduced as a multi-nodal framework built in the open-source programming language Julia, emphasising accessibility, transparency, and ease of expansion through collaboration ^45^. Together, these initiatives highlight the ongoing efforts within the energy research community to advance open science practices and improve the credibility and impact of their work.

As pointed out in the previous section, energy research is challenging to comprehend without specific education. Therefore, open educational resources that reach a larger society share can facilitate research dissemination. Universal access to education is becoming more common in the energy domain, with Open Courseware uploaded to platforms like YouTube or Coursera. In particular, we observe an increase in open-source material linked with open educational resources. This is the case of OSeMOSYS ^2^, an open-source energy system model that provides multiple tutorials, interfaces, and documentation for new users.

## V. Conclusion

This essay presents and discusses the benefits, pitfalls and practices of open science and, in particular, its application in the energy domain. In general, open science fosters accessibility, transparency, and reproducibility across all research stages. The sharing of methods, data and knowledge can promote collaboration while mitigating the duplication of work, thereby saving time and costs and accelerating the progress of science. At the individual level, researchers who make their publications and data publicly accessible typically experience an increase in citations, enhancing their visibility and impact.

Despite these benefits, significant barriers persist, hindering researchers from fully adopting the principles of open science. Some ethical and legal conflicts like patient privacy protection are well-founded and require careful consideration. Conversely, others arise from researchers’ perceived risks, such as the potential loss of competitive advantage or the ineffective use of time and resources. Addressing and discussing these concerns is crucial to enabling wider adoption of open science practices.

Specifically, energy research is an applied field with significant societal and policy implications in the economy, the environment, the progress of societies and geopolitics. As such, it is a domain that can benefit from the transparency and collaboration that open science can foster. By adhering to open science principles, energy researchers can build trust among policymakers and the general public while promoting data-based decision-making. Moreover, there has been a marked increase in adherence to open science, particularly concerning open-source initiatives and open-data platforms. However, open science faces considerable barriers among energy researchers. The complexities inherent in the energy sector pose substantial obstacles to its implementation, including intellectual property concerns, intricate models and technologies, interdisciplinary barriers, and infrastructure security issues.

Open science brings undeniable benefits to the entire research community, including those within the energy domain. Initiatives such as the FAIR principles are key in guiding researchers toward adopting appropriate methodologies that can promote the research ethos of universalism and communism. At the same time, recognising the limitations and perceived barriers of open science can help researchers evaluate which methodologies apply to their specific contexts and alleviate apprehensions to unlock the advantages of disseminating their work to a broader audience.

## Ethics and consent

Ethical approval and consent were not required.

## Data Availability

No data are associated with this article.

## Footnotes

^1^ Algorithms and models whose underlying math and methods are hidden or too complex to explain. With black-box models, we know the input and the output, but we cannot easily understand how the output was generated.

^2^ http://www.osemosys.org/
