# Supplementary material for: Open science in energy research
Source: Open Res Eur. 2025 Jan 22;3:50. Originally published 2023 Mar 31. [Version 2] doi: 10.12688/openreseurope.15707.2 (PMC11907185; doi:10.12688/openreseurope.15707.2)
Supplement: Peer review history

# Reviewer report: Reviewer response for version 2

**DOI:** 10.21956/openreseurope.21004.r50172

**Published:** 2025-03-22

**Authors:** Stephan Ferenz

**Affiliations:** 1 University of Oldenburg, Oldenburg, Germany

- The article gives an overview of the different aspects of Open Science and attempts to show the current state of Open Science in Energy Research.
- The introduction nicely outlines the latest developments to Open Science and its relevance. It motivates why the state of Open Science in energy research is an important topic and relevant to analyze.
- The second section gives a good overview about different aspects of Open Science.
- The third section also addresses the general topic of Open Science and outlines different barriers of  Open Science. This give an good overview of different barriers and short comings of Open Science.
- Finally, the fourth section analyzes Open Science in energy research. Especially, the barriers part can be improved by reusing the categorization of barriers for Open Science from section III.
- The last section of the paper discusses the different aspects of Open Science and outlines advantages and disadvantages. We think the paper would profit from an outlook section which could point out what additional research is needed to get a better overview of the state of Open Science in energy research.

Major Issues

1. Definition of Research Software (p. 4): The common definition for research software is by Gruenpeter et al. ( https://zenodo.org/records/5504016): “Research Software includes source code files, algorithms, scripts, computational workflows and executables that were created during the research process or for a research purpose.”
2.  IV-B “Barriers and limitations of open science in energy research” does not link to the results of the section III “Barriers and shortcomings of open science”.
3. Especially the initiatives (IV-C) could be better analyzed regarding which part of open science they address/contribute to (e.g., by adding a table).

Minor Issues

1. Page 4: “of scientific literature. . There are two main paths“ There are two periods instead of one.
2. Page 4: “With open science, the research practices by inducing the reproducibility of research.“ This sentence misses a verb.
3. Page 4.: “In an attempt to answer some of these questions and enhance the practices of researchers, Wilkinson et al. propose the publications enhancesFAIR principles to guide stakeholders on how to manage and steward their research data effectively.” “publication enhances” seems misplaced.
4. Page 6: “This situation demonstrates that promoting open data is feasible if effective data management protocols are prioritised in energy research projects, aligning with the growing trend of open access initiatives in Europe.Making knowledge accessible through open access increases dissemination and, consequently, the presence of scientific arguments in social debates and cooperation between policy and research.” A space is missing before the new sentence.

Where applicable, are recommendations and next steps explained clearly for others to follow? (Please consider whether others in the research community would be able to implement guidelines or recommendations and/or constructively engage in the debate)

No

Does the article adequately reference differing views and opinions?

Partly

Are all factual statements correct, and are statements and arguments made adequately supported by citations?

Partly

Is the rationale for the Open Letter provided in sufficient detail? (Please consider whether existing challenges in the field are outlined clearly and whether the purpose of the letter is explained)

Yes

Is the Open Letter written in accessible language? (Please consider whether all subject-specific terms, concepts and abbreviations are explained)

Yes

Reviewer Expertise:

Stephan Ferenz: Research data management and Open Science in Energy Research.

I confirm that I have read this submission and believe that I have an appropriate level of expertise to confirm that it is of an acceptable scientific standard, however I have significant reservations, as outlined above.

# Reviewer report: Reviewer response for version 2

**DOI:** 10.21956/openreseurope.21004.r51917

**Published:** 2025-03-21

**Authors:** Antonio F. Rodriguez-Matas

**Affiliations:** 1 Universidad Pontificia Comillas, Madrid, Spain

I believe this is an important and timely topic that deserves attention. The authors have done a good job of synthesizing the current landscape of open science practices and their application to energy research. However, I've identified some areas that could be enhanced or further addressed:

- Limitations of Open peer review: The manuscript presents open peer review as a potential solution without fully acknowledging its limitations. When reviewers aren't anonymous, there's a real risk that reviews might become less candid due to fears of potential backlash, especially for early-career researchers reviewing work by established figures, or in research areas where the number of researchers is not that large. Consider presenting a more balanced discussion of peer review models.

 

- Open Access and predatory publishing: Open access is proposed as a solution to increase dissemination, but it very much overlooks the problem of predatory journals. These questionable publications exploit open-access models while undermining scientific integrity. I would suggest including some analysis of how predatory publishing threatens the open-access model and potential solutions like better journal vetting and institutional support for legitimate open-access venues.

 

- Recognizing "negative" results: When discussing questionable research practices, one issue not adequately considered is the scientific community's reluctance to publish "negative" results, which creates incentives for these questionable research practices. You can find literature about the bias against publishing null findings that drives researchers toward selective reporting (e.g., Fanelli (2012)). Publishing negative results would share valuable knowledge about approaches that didn't work, save resources across the scientific community, prevent others from wasting time on dead-end approaches, and recognize the work and resources of researchers with publications and citations. Therefore, I suggest discussing how open science could help create space for negative results through dedicated journal sections or data repositories.

 

- Alternative metrics concerns: The statement about using altimetric like social media mentions to evaluate research needs more critical analysis. Measuring scientific quality through Twitter mentions or blog posts is problematic. There is also literature about how altmetrics often measure attention rather than quality, with sensationalistic studies getting more online attention regardless of their rigor or real impact.

 

- Incentive structures and Open science implementation: A fundamental issue that deserves more attention is how current academic incentive structures could work against open science adoption. The manuscript mentions time constraints but doesn't fully analyze how evaluation systems could fail to reward open science practices. The paper notes that open science requires "additional effort and time consumption" from researchers making their work open, while others benefit by saving time and resources. This could create an asymmetric situation where those practicing open science bear costs while others reap rewards. In an academic system where researchers compete for positions and funding based on publication metrics, there's little incentive to help competitors without recognition. I would suggest to examine how academic evaluation criteria create incentives against open science, the possibilities for changing these incentive structures (e.g., assessment indicators that might prioritize collaboration over competition, specific proposals for better aligning individual researcher incentives with the collective benefits of open science, etc).

In conclusion, while the manuscript makes a valuable contribution to understanding open science in energy research, addressing these points would strengthen its analysis and practical value.

Where applicable, are recommendations and next steps explained clearly for others to follow? (Please consider whether others in the research community would be able to implement guidelines or recommendations and/or constructively engage in the debate)

Yes

Does the article adequately reference differing views and opinions?

Partly

Are all factual statements correct, and are statements and arguments made adequately supported by citations?

Partly

Is the rationale for the Open Letter provided in sufficient detail? (Please consider whether existing challenges in the field are outlined clearly and whether the purpose of the letter is explained)

Yes

Is the Open Letter written in accessible language? (Please consider whether all subject-specific terms, concepts and abbreviations are explained)

Yes

Reviewer Expertise:

Open-source modeling, energy systems analysis, robust decision-making.

I confirm that I have read this submission and believe that I have an appropriate level of expertise to confirm that it is of an acceptable scientific standard, however I have significant reservations, as outlined above.

## References

[1] : Negative results are disappearing from most disciplines and countries. *Scientometrics* .2012;90(3) : 10.1007/s11192-011-0494-7 891-904 10.1007/s11192-011-0494-7

# Reviewer report: Reviewer response for version 2

**DOI:** 10.21956/openreseurope.21004.r51919

**Published:** 2025-03-20

**Authors:** John Draper

**Affiliations:** 1 Nonkilling Economics and Business Research Committee, Center for Global Nonkilling, Hawaii, USA

I have presumably been invited to review this open letter owing to my co-authored work on economic and geopolitical theory regarding open science in nuclear fusion, including the application of Mertonian Norms, the rise of the fusion energy research (FER) private sector and the growing phenomenon of private sector patenting of fusion pathways, and the diplomatic aspects of FER. Consequently, I will largely limit myself to comments on this field. So, to begin, I would argue that fusion energy FER be referenced in this letter, because a net energy gain was achieved last year, because a fusion energy research private sector is now well established, and because of the (public sector funded) quasi-open FER’s diplomatic role in peacebuilding, less now because of private sector progress, but still important in that key breakthroughs should be made for all humanity – as if not, they will simply be copied, with IP ignored, and become a source of conflict.

 With fusion, it is presently likely that the first commercial prototype will be rooted in the public sector funded tokamak. However, having historical public sector roots does not equal being open now, and the MIT-originated Commonwealth Fusion Systems, whilst maintaining the spirit of openness with its approach to building a tokamak fusion reactor, e.g., by publishing key developments, is very much a commercial development. It is also possible that the fusion technology pathway that becomes the most commercially viable becomes very much closed. As in the case of Betamax and VHS, because of the bandwagon effect, it is not necessarily the case that what is superior in terms of quality in the field of fusion will win the competition in the long term. In this scenario, the FER market is influenced by different actors through market behaviour and patenting behaviour in ways such that an inferior, but better backed product predominates. This phenomenon was also visible in fission, e.g., in the way thorium was shut down in the 1960s-1970s. Bandwagoning can also occur with public sector (quasi-open) science fusion, and it can be related to the sunk cost fallacy, most notably as ITER has attracted huge amounts of funding into tokamak research, at the expense of other technology pathways. What is clear from such experiences is that there has to be an optimum balance in the ratio of (mainly public sector-funded) open science and private sector commercial development of FER, and that complex economic modelling may help.

 As the Fusion Diffusion Team of George Washington University’s Business School’s European Union Research Center argue elsewhere, in FER what is key is that certain foundational scientific aspects of FER, such as tritium fuel production, neutron resistant materials development, and fuel cycle research, remain as open as possible, with the technical development from concept through to prototype of the exact technique, whether from the magnetic confinement or inertial confinement families, or another, being the private sector icing on the cake. Thus, it is necessary for there to be at least partially open science FER, in the form of public support for next generation fusion reactors, through e.g., US DoE ARPA-e programs and the INFUSE program, as it may be these next-level machines that will develop new stable plasma geometries.

 Regarding the diplomatic benefits of open science energy research, while regional (e.g., EU) and international cooperation are mentioned in the article, science diplomacy (and peacebuilding) is not specifically mentioned and should be. In terms of FER, the case of ITER is well known, and I provide (variously formatted) references below for options for future FER open diplomacy, including a Global Commission to Accelerate Fusion Energy (GCAFE). Again, one of the main concerns of fusion is that if a fusion technology pathway is commercialisable and particularly well protected by patenting, for instance through patent thicketing, if the price of a reactor is too high, the majority of the world’s countries will simply buy a reverse engineered version from a third party country, most obviously China. The only way we see to mitigate that is to ensure co-development by Global South countries at both the open science stage and at the commercialising stage, through individual country, regional or thematic bloc (e.g., ASEAN, OPEC), and even G77 participation and investment investment, i.e., the GCAFE route. In this way, open energy science diplomacy could safeguard intellectual property democratically, by offering the Global South a place at the table to co-develop FER, positively impacting education and development, and opening up the possibility for countries to operate and maintain their own fusion plants – much as was achieved with the US’s Atoms for Peace approach, at least with regard to scientific machines.

 To conclude, I am not advocating a new section of the open letter (or essay?). Given the historical importance of EU, UK, and US DoE open science to FER, I do suggest a few mentions of it along the lines presented above, including that FER presents an interesting case study as it is transitioning to private sector, that open science FER helps offer a more open playing field in the face of bandwagoning and is very much still required in core areas of fusion energy development, like the tritium cycle, and that FER science diplomacy may be the only way to prevent a US-China FER war.

 Specifically in response to the following question: “Is the rationale for the Open Letter provided in sufficient detail? (Please consider whether existing challenges in the field are outlined clearly and whether the purpose of the letter is explained)”.

 I feel that mentioning the multiple challenges facing FER is worth referring to as it transitions from planning for the ITER phase to DEMO(nstration) phase planning.

 In response to this question: “Is the Open Letter written in accessible language? (Please consider whether all subject-specific terms, concepts and abbreviations are explained)”

 I would say that the letter requires proper proofreading, as there are errors throughout. So, for instance, “These practices raise ethical concerns not only stop the dissemination of research but also hinder the identification of research misconduct.” should read “These practices raise ethical concerns **in that they** not only stop the dissemination of research but also hinder the identification of research misconduct.”

 I would also say that in this sentence the word in bold should be reworded: “Some ethical and legal conflicts like **patient** → **patent** privacy protection are well-founded and require careful consideration.”

 In response to the following question: “Where applicable, are recommendations and next steps explained clearly for others to follow? (Please consider whether others in the research community would be able to implement guidelines or recommendations and/or constructively engage in the debate)”

 I would say that recommendations specific to some challenges for humanity should be mentioned. In the energy sphere, it is clear from IRENA’s Global Commission on the Geopolitics of the Energy Transformation that it is possible that a small number of countries will through patenting achieve energy technology dominance, in a way that will “stifle competition, suppress innovation, and distort markets”. Then, how do patents facilitate or restrict the Green Climate Fund aspect of the Paris Agreement re the role of the private sector and country ownership of GCF projects? How is WIPO managing its green energy obligations (WIPO GREEN)?

 So, for an instance of one specific energy research sector, in a post-ITER world, what international mechanisms or vehicles are available for balancing Global North-South open science diplomacy-driven co-development against private sector development in FER? One option cited is the global commission. Certainly, open science would seem to have a role to play in implementing the recommendations of the International Energy Agency’s (IEA’s) Global Commission on People-Centred Clean Energy Transitions, such as the elimination of energy poverty, and in the IEA’s recommendations of the Global Commission for Urgent Action on Energy Efficiency, such as that for greater international collaboration. The authors do not need to provide detailed considerations of these topics, but the open letter may benefit from alluding to them.

 Relevant reading (variously formatted):

 Carayannis, Elias; Draper, John; and Bhaneja, Balwant (2022) "Fusion Energy for Peacebuilding: A Trinity Test-Level Critical Juncture," *Peace and Conflict Studies*: Vol. 29: No. 1, Article 4.

 Available at: https://nsuworks.nova.edu/pcs/vol29/iss1/4- This reference highlights FER as a global challenge with high symbolic importance for peacebuilding, one of the potential applications of open energy science via the UNSDG framework, specifically UNSDG 16 (peacebuilding).

 Todd K. Harding, Melanie J. Khanna, and Raymond L. Orbach, “International Fusion Energy

 Cooperation: ITER as a Case Study in Science and Diplomacy,” Science & Diplomacy, Vol.

 1, No. 1 (March 2012). http://www.sciencediplomacy.org/article/2012/international-fusion-energy-cooperation-This reference presents ITER as a case study in quasi-open energy science diplomacy.

 Also ref 1,2,3,4

 This reference applies the Mertonian Norms to FER-ref 1

 This reference applies the global commission concept to FER to balance Global North-South relations, including the management of open (for education and *co-development* of energy) versus private sector requirements against a UNSDG framework-Ref-2

 This reference updates Todd et al. and highlights ITER as an example of energy science diplomacy-Ref-3

 This reference highlights the transition from quasi-open (public sector) FER to private-sector FER through a study of patenting-Ref-4

Where applicable, are recommendations and next steps explained clearly for others to follow? (Please consider whether others in the research community would be able to implement guidelines or recommendations and/or constructively engage in the debate)

Partly

Does the article adequately reference differing views and opinions?

Yes

Are all factual statements correct, and are statements and arguments made adequately supported by citations?

Yes

Is the rationale for the Open Letter provided in sufficient detail? (Please consider whether existing challenges in the field are outlined clearly and whether the purpose of the letter is explained)

Partly

Is the Open Letter written in accessible language? (Please consider whether all subject-specific terms, concepts and abbreviations are explained)

Partly

Reviewer Expertise:

Geopolitics of nuclear fusion, including for peacebuilding

I confirm that I have read this submission and believe that I have an appropriate level of expertise to confirm that it is of an acceptable scientific standard, however I have significant reservations, as outlined above.

## References

[1] : The economic logic of open science in fusion energy research: A policy perspective. *Energy Policy* .2024;186: 10.1016/j.enpol.2024.113983 10.1016/j.enpol.2024.113983

[2] : Democratic Engagement in Sustainable Energy Innovation: Applying the Quintuple Innovation Helix to Manage Accelerating Fusion Energy Through an IEA-Backed Global Commission. *IEEE Transactions on Engineering Management* .2024;71: 10.1109/TEM.2024.3428975 14293-14306 10.1109/TEM.2024.3428975

[3] : The place of peace in the ITER machine assembly launch: Thematic analysis of the political speeches in the world’s largest science diplomacy experiment. *Peace and Conflict: Journal of Peace Psychology* .2021;27(4) : 10.1037/pac0000559 665-668 10.1037/pac0000559

[4] : The growth of intellectual property ownership in the private-sector fusion industry. *Fusion Engineering and Design* .2021;173: 10.1016/j.fusengdes.2021.112815 10.1016/j.fusengdes.2021.112815

# Reviewer report: Reviewer response for version 2

**DOI:** 10.21956/openreseurope.21004.r50559

**Published:** 2025-03-13

**Authors:** Vinod Veeramachaneni

**Affiliations:** 1 Colorado Technical University, Colorado Springs, USA

**Summary of the Article**

 The paper examines the role of **open science** in energy research, focusing on its principles, benefits, and challenges. It highlights the **barriers posed by proprietary data, closed-access publishing, and black-box models** that hinder transparency and reproducibility in the field. The article advocates for **greater openness in research methodologies, data sharing, peer review, and publication models** while discussing potential drawbacks such as ethical concerns, competitive disadvantages, and the complexities of implementing open science. The paper also addresses current initiatives promoting open science, including **open-source platforms, FAIR principles, and Horizon 2020 policies.**

 The discussion is **well-structured**, progressing logically from definitions and principles of open science to its implications in energy research. The authors present a **balanced view**, acknowledging both the positive impact of open science and the barriers to its adoption.

**Assessment of Key Review Criteria**

**1. Is the rationale for the Open Letter provided in sufficient detail?**

**Answer: Partly**

 While the paper discusses challenges in the field, the rationale for the **Open Letter** is not clearly articulated. The authors outline issues related to **closed research models, proprietary data, and limited access to scientific knowledge**, but they do not explicitly state:

- **Why an Open Letter is necessary** at this stage.
- **Who the primary audience is** (e.g., policymakers, funding agencies, academic institutions).
- **What specific action the Open Letter aims to encourage** beyond general support for open science.

**Suggested Improvements:**

- Provide a clearer explanation of **why** an Open Letter is the chosen format to advocate for open science in energy research.
- Specify **the intended recipients** and what response or policy change the letter seeks to achieve.
- Reference **similar initiatives** (if any) and their outcomes to strengthen the argument.

**2. Does the article adequately reference differing views and opinions?**

**Answer: Partly**

 The article acknowledges **challenges and criticisms** of open science, such as:

- **Legal and ethical concerns** related to data privacy.
- **Competitive disadvantages** for researchers who share their work openly.
- **Potential quality concerns** due to open-access publishing and the lack of editorial oversight in preprint repositories.

However, the discussion does not fully explore **counterarguments from stakeholders who oppose open science outright** or perspectives from:

- **Industry representatives** who may rely on proprietary research for commercial purposes.
- **Policymakers or funding agencies** that impose restrictions on data sharing.
- **Academics in fields with lower adoption rates of open science** and their reasoning.

**Suggested Improvements:**

- Incorporate viewpoints from **opponents of open science**, citing studies or examples where open science has been contested.
- Provide **specific examples** of institutions or researchers who have raised concerns about open science adoption.
- Discuss possible **compromises or hybrid models** that balance openness with competitive concerns.

**3. Is the article scientifically sound?**

**Answer: Yes, with minor revisions**

 The article is **well-researched and supported by relevant literature**. However, minor improvements are needed in:

1. **Clarifying Definitions:**
  - The **Open Letter** rationale should be explicitly stated.
  - Some **technical discussions (FAIR principles, metadata)** could be made more accessible.
2. **Strengthening the Discussion on Open Science Initiatives in Energy Research:**
  - The paper discusses **OpenMod, Horizon 2020, and European open-access initiatives**, but lacks specific examples of **successful implementation in energy research**.
  - Providing **case studies** where open science has led to improved transparency, collaboration, or policy impact would enhance the discussion.
3. **Addressing Ethical and Security Concerns in More Depth:**
  - The **privacy risks of sharing sensitive energy data** are mentioned but not fully explored.
  - There should be more discussion on **how researchers can navigate legal restrictions** while maintaining openness.

**Final Recommendation**

 The paper makes a **valuable contribution** to the discussion on open science in energy research. While it is generally **well-structured and well-supported**, the authors should address the following points before final publication:

1. **Clearly define the purpose of the Open Letter**, including its target audience and intended impact.
2. **Expand on differing opinions**, particularly from industry and policymakers who may resist open science initiatives.
3. **Enhance the discussion of case studies** where open science has been successfully implemented in energy research.
4. **Clarify ethical and legal concerns**, including potential solutions for handling sensitive data.

Where applicable, are recommendations and next steps explained clearly for others to follow? (Please consider whether others in the research community would be able to implement guidelines or recommendations and/or constructively engage in the debate)

Yes

Does the article adequately reference differing views and opinions?

Yes

Are all factual statements correct, and are statements and arguments made adequately supported by citations?

Yes

Is the rationale for the Open Letter provided in sufficient detail? (Please consider whether existing challenges in the field are outlined clearly and whether the purpose of the letter is explained)

Partly

Is the Open Letter written in accessible language? (Please consider whether all subject-specific terms, concepts and abbreviations are explained)

Yes

Reviewer Expertise:

Decentralized Trust Management in Web 3.0 – Focusing on trust models without central authority in DeFi, decentralized identity, and governance.Artificial Intelligence and Software Engineering – Applying AI to optimize software development, DevOps, and Agile methodologies.Cybersecurity and Zero Trust – Researching IAM models, authentication mechanisms, and blockchain for security.Cloud Computing and Edge Computing – Studying decentralized platforms, latency reduction, and IoT integration.Trading Applications and Financial Technology – Building microservices and scalable system architectures for trading platforms.Open Science and Research Transparency – Advocating for open access, reproducibility, and data-sharing practices in scientific research.Energy Research and Digital Transformation – Exploring open science principles in energy policy, sustainability, and modeling

I confirm that I have read this submission and believe that I have an appropriate level of expertise to confirm that it is of an acceptable scientific standard.

# Reviewer report: Reviewer response for version 2

**DOI:** 10.21956/openreseurope.21004.r50264

**Published:** 2025-02-28

**Authors:** Nicki Lisa Cole

**Affiliations:** 1 Open and Reproducible Research Group, Graz, Austria

Thank you to the authors for the opportunity to review this short paper. I believe it has promise to be useful to those working in energy research, however in its current state, I believe the manuscript has several areas that require improvement. There are three key issues for me.

 1. A lengthy description of open science that is completely disconnected from the state of the art on this topic. For example, the paper's articulation of what open science is ignores the now gold standard definition of open science provided by the UNESCO Recommendation on Open Science, and therefore leaves out key aspects of open science entirely (open dialogue with other knowledge systems, open science infrastructures, and open engagement of societal actors). I believe that this critical issue can be resolved by significantly shortening the first two section of the paper to provide a simple, succinct definition of open science and specifying the aspects that it finds relevant to the energy research community. The paper could simply point to the extensive literature on this topic rather than trying to encapsulate it in three pages. Further, this would serve to focus the paper on what it claims to focus on: open science in energy research (rather than having a three-page, dense and flawed background section prior to getting to the point).

 2. There is a lack of engagement with the robust evidence available that documents the academic and societal impact of open science that would be relevant to discuss in relation to energy research, particularly in section IV.A, which relies on anecdotal evidence rather than published evidence in the first few paragraphs. See recent reviews by Klebel et. al. (https://osf.io/preprints/socarxiv/ptjub_v1) and Cole et al. Ref: 1 to find relevant sources to integrate into your discussion. Otherwise, I find the second half of the paper, focused on open science in energy research, to be generally strong and well done.

 3. There are some issues with language throughout the paper. Some bizarre phrasing caught my eye, and suggests either the use of a translation and/or AI tool. For example, the second sentence of the paper, which uses the phrase "enticing the arcane and occult information to a selected group." I have no idea what this should mean. I also find examples of incorrect word usage, like "prone" in the third paragraph of the paper, and typos throughout. We recommend professional editing

 I'll make one more criticism, which I find to be more minor than the others, given that I have already suggested cutting the first half of the paper significantly. There are some logical errors in the argumentation which reflect a lack of engagement with the state of the art on open science. On page 4, the authors claim that open science "can prevent undesirable research practices by inducing [word choice] transparency, debate, and reproducibility of research." In fact, there is scant and mixed evidence to this effect. This claim is not backed by evidence. The related reference to and discussion of "inappropriate research practices" is also troublingly disconnected from the robust literature on research integrity and questionable research practices.

 I hope the authors will take this critical feedback in the constructive spirit in which it is offered, and proceed with revisions that bring the energy aspect more clearly into focus as the heart of the paper.

Where applicable, are recommendations and next steps explained clearly for others to follow? (Please consider whether others in the research community would be able to implement guidelines or recommendations and/or constructively engage in the debate)

Not applicable

Does the article adequately reference differing views and opinions?

Partly

Are all factual statements correct, and are statements and arguments made adequately supported by citations?

Partly

Is the rationale for the Open Letter provided in sufficient detail? (Please consider whether existing challenges in the field are outlined clearly and whether the purpose of the letter is explained)

Partly

Is the Open Letter written in accessible language? (Please consider whether all subject-specific terms, concepts and abbreviations are explained)

Partly

Reviewer Expertise:

Metascience, open science, reproducibility

I confirm that I have read this submission and believe that I have an appropriate level of expertise to state that I do not consider it to be of an acceptable scientific standard, for reasons outlined above.

## References

[1] : The societal impact of Open Science: a scoping review. *R Soc Open Sci* .2024;11(6) : 10.1098/rsos.240286 240286 10.1098/rsos.240286 39100167 PMC11296153

# Reviewer report: Reviewer response for version 2

**DOI:** 10.21956/openreseurope.21004.r51306

**Published:** 2025-02-27

**Authors:** Zhaoyun Zeng

**Affiliations:** 1 Argonne National Laboratory, Lemont, Illinois, USA

This letter provides a nice short review of the concept of open science and clearly outlines the importance of open science to energy research.

 Here are some minor revision suggestions:

 1. “5. Open source promotes the dissemination of research software like code.” What does “like code” refer to? Revise this expression.

 2. “Pfenninger *et al.*34 identify three other beneficial outcomes of adhering to open science in energy research” Here, it should be “identified”.

 3. “This situation demonstrates that promoting open data is feasible if effective data management protocols are prioritised in energy research projects, aligning with the growing trend of open access initiatives in Europe. Making knowledge accessible through open access increases dissemination and, consequently, the presence of scientific arguments in social debates and cooperation between policy and research.” A space needs to be added between the two sentences.

 4. The following review is about the limited accessibility of suitable future weather data for energy research. It can be included in the discussions of open data (Refer 1).

Where applicable, are recommendations and next steps explained clearly for others to follow? (Please consider whether others in the research community would be able to implement guidelines or recommendations and/or constructively engage in the debate)

Yes

Does the article adequately reference differing views and opinions?

Yes

Are all factual statements correct, and are statements and arguments made adequately supported by citations?

Yes

Is the rationale for the Open Letter provided in sufficient detail? (Please consider whether existing challenges in the field are outlined clearly and whether the purpose of the letter is explained)

Yes

Is the Open Letter written in accessible language? (Please consider whether all subject-specific terms, concepts and abbreviations are explained)

Yes

Reviewer Expertise:

Building energy modeling, climate change

I confirm that I have read this submission and believe that I have an appropriate level of expertise to confirm that it is of an acceptable scientific standard, however I have significant reservations, as outlined above.

## References

[1] : A review of future weather data for assessing climate change impacts on buildings and energy systems. *Renewable and Sustainable Energy Reviews* .2025;212: 10.1016/j.rser.2024.115213 10.1016/j.rser.2024.115213

# Reviewer report: Reviewer response for version 2

**DOI:** 10.21956/openreseurope.21004.r50561

**Published:** 2025-02-10

**Authors:** Ronald Marquez

**Affiliations:** 1 University of Girona, Girona, Spain

The authors connect classical concepts of scientific ethos (e.g., those proposed by Merton) with modern open science initiatives. The manuscript’s exploration of ethical and legal dimensions—particularly around intellectual property (IP) rights—offers insights into the challenges that researchers and industry partners face when attempting to implement open research practices.

 Areas for Enhancement

 1. Quantitative Data: Although the manuscript references studies indicating increased citations for open data publications, additional or more up-to-date datasets—especially on global vs. regional open research adoption—would strengthen the paper’s evidence base and support deeper trend analysis. Particularly, trends on open research of Europe vs. other regions such as Asia or Middle East and discuss if open research in Europe gives (or not) advantage to regions that prioritize industrial research (not open).

 2. Industry Case Studies: The discussion on private-sector uptake would benefit from concrete examples illustrating how specific energy companies or consortia have successfully integrated open research practices (or, conversely, have declined to do so). It is importante to include trends on the bigger players in energy, are they willing to make open research? Is that on their advantage? What could governments or institutions provide them as advantage to decide for an open research route?

 3. AI Potential and Limitations: A broad discussion on AI implementation is important. Universities are falling behind on computational power vs. private companies and have started making public-private partnerships. 

 The California State University (CSU) has announced a landmark public-private initiative with leading tech companies, including Adobe, Alphabet (Google), AWS, IBM, Instructure, Intel, LinkedIn, Microsoft, NVIDIA, OpenAI, and the Office of California Governor Gavin Newsom. This collaboration aims to establish CSU as the nation's first and largest AI-powered university system, providing AI tools and training across all 23 campuses to benefit over 460,000 students and 63,000 faculty and staff. The initiative seeks to enhance education and research, preparing the CSU community to meet the evolving demands of California's workforce and economy.

Where applicable, are recommendations and next steps explained clearly for others to follow? (Please consider whether others in the research community would be able to implement guidelines or recommendations and/or constructively engage in the debate)

Yes

Does the article adequately reference differing views and opinions?

Yes

Are all factual statements correct, and are statements and arguments made adequately supported by citations?

Yes

Is the rationale for the Open Letter provided in sufficient detail? (Please consider whether existing challenges in the field are outlined clearly and whether the purpose of the letter is explained)

Yes

Is the Open Letter written in accessible language? (Please consider whether all subject-specific terms, concepts and abbreviations are explained)

Yes

Reviewer Expertise:

Energy, bioenergy, biofuels, emulsions, interfaces, lignocellulosic materials

I confirm that I have read this submission and believe that I have an appropriate level of expertise to confirm that it is of an acceptable scientific standard, however I have significant reservations, as outlined above.

# Reviewer report: Reviewer response for version 1

**DOI:** 10.21956/openreseurope.16970.r31988

**Published:** 2023-07-26

**Authors:** Gesche Huebner

**Affiliations:** 1 University College London, London, England, UK

I overall much enjoyed reading this open letter; open science and ethics in energy research important and overlooked aspect. The authors describe what open science is and discuss its benefits and drawbacks. They then focus on the energy research sector and show how it lags behind other disciplines

 I have four points I suggest the authors to engage with:

1. In the discussion of open data, I would expect to see the FAIR principles mentioned, i.e. Findability, Accessibility, Interoperability, and Reusability, see e.g. here: https://www.nature.com/articles/sdata201618. Also, adding some citations around the benefits of open data would be appropriate (e.g. https://journals.plos.org/plosone/article?id=10.1371/journal.pone.0230416 but there are others).
2. As a reviewer, it is always awkward pointing to your own publications but myself and colleagues have published previously around openness and transparency in energy research, reflecting on many of the difficulties mentioned by the authors of this paper, such as the heterogeneity of energy research and the quick changes in the energy landscape. We would encourage the authors to engage with our publications, in particular this peer-reviewed journal article:  Huebner, GM; Fell, MJ; Watson, NE; (2021) Improving energy research practices: guidance for transparency, reproducibility and quality. **Buildings & Cities** , 2 (1) pp. 1-20. 10.5334/bc.67.
3. The discussion of the publishing industry revenue structure could be given more depth. There have been quite a lot of changes, see e.g. here for a discussion on Plan S ( https://www.nature.com/articles/d41586-021-00883-6); there are different levels of open access (Gold vs. Green) etc. Also, I cannot quite follow the argument how creating different streams of revenue would aid open access. Many journals already are open access or have an open access option (even subscription journals). Is the idea here to replace the subscription model with an open access model through payment for individual services instead of a university subscribing to a journal / publisher? Could there be unintended consequences especially for more junior scholars / those not holding large grants?
4. About preprints being potentially problematic because of the lack of oversight, this is a fair point – but they bring many benefits (e.g. earlier and wider access to scientific findings; possibility to include not yet published work in grant applications by linking to a preprint) and it might be an option to simply shape a mindset that preprints come with a ‘health warning’. E.g. preprint servers could overlay all preprints with a clear message saying “not peer-reviewed, no assessment of validity and reliability done” and the onus would be on the reader to interpret them as preliminary.

Finally, the paper nicely lays out how energy research is heterogeneous but then it does focus in particular on energy systems modelling and its open modelling initiative. Whilst this is an incredibly important topic, energy research is much broader and some more nuance around this might be nice to add.

Where applicable, are recommendations and next steps explained clearly for others to follow? (Please consider whether others in the research community would be able to implement guidelines or recommendations and/or constructively engage in the debate)

Not applicable

Does the article adequately reference differing views and opinions?

Yes

Are all factual statements correct, and are statements and arguments made adequately supported by citations?

Partly

Is the rationale for the Open Letter provided in sufficient detail? (Please consider whether existing challenges in the field are outlined clearly and whether the purpose of the letter is explained)

Yes

Is the Open Letter written in accessible language? (Please consider whether all subject-specific terms, concepts and abbreviations are explained)

Yes

Reviewer Expertise:

energy research; open science

I confirm that I have read this submission and believe that I have an appropriate level of expertise to confirm that it is of an acceptable scientific standard, however I have significant reservations, as outlined above.

## References

[1] : The FAIR Guiding Principles for scientific data management and stewardship. *Sci Data* .2016;3: 10.1038/sdata.2016.18 160018 10.1038/sdata.2016.18 26978244 PMC4792175

[2] : The citation advantage of linking publications to research data. *PLoS One* .2020;15(4) : 10.1371/journal.pone.0230416 e0230416 10.1371/journal.pone.0230416 32320428 PMC7176083

[3] : Improving energy research practices: guidance for transparency, reproducibility and quality. *Buildings and Cities* .2021;2(1) : 10.5334/bc.67 1-20 10.5334/bc.67

[4] : A guide to Plan S: the open-access initiative shaking up science publishing. *Nature* .2021; 10.1038/d41586-021-00883-6 10.1038/d41586-021-00883-6 33833458

# Response

**Published:** 2025-01-14

**Authors:** Raquel Alonso Pedrero

**Affiliations:** Norwegian University of Science and Technology, Norway

We are very grateful for your comments and positive feedback. Please find below your feedback, followed by our replies.

**Reviewer Comments:**

**General Comments:** 

 I overall much enjoyed reading this open letter; open science and ethics in energy research important and overlooked aspect. The authors describe what open science is and discuss its benefits and drawbacks. They then focus on the energy research sector and show how it lags behind other disciplines   

 I have four points I suggest the authors to engage with: 

**Author Response:**

 We are very glad to know that you found the topic interesting and meaningful. Also, we are grateful to get your feedback as it improved the quality of the new manuscript. Each comment is addressed below. 

**Comment 1:**

 In the discussion of open data, I would expect to see the FAIR principles mentioned, i.e. Findability, Accessibility, Interoperability, and Reusability, see e.g. here: https://www.nature.com/articles/sdata201618. Also, adding some citations around the benefits of open data would be appropriate (e.g. https://journals.plos.org/plosone/article?id=10.1371/journal.pone.0230416 but there are others). 

**Author Response:**

 Thank you. We agree that the FAIR principles are essential when discussing open science. We revised Section II to reflect this. In the subsection “C. Applying open science”, focused on the integration of open science in research, we introduce the FAIR principles.   Moreover, the new description of the key principles of open science now includes citations as one of the benefits of open data, including the citation to the suggested article. 

**Comment 2:**

 As a reviewer, it is always awkward pointing to your own publications but myself and colleagues have published previously around openness and transparency in energy research, reflecting on many of the difficulties mentioned by the authors of this paper, such as the heterogeneity of energy research and the quick changes in the energy landscape. We would encourage the authors to engage with our publications, in particular this peer-reviewed journal article:  Huebner, GM; Fell, MJ; Watson, NE; (2021) Improving energy research practices: guidance for transparency, reproducibility and quality. Buildings & Cities , 2 (1) pp. 1-20. 10.5334/bc.67. 

**Author Response:**

 Thank you for recommending your paper, it was a definitely a publication that we needed to read. We enjoyed the discussion, and the approach proposed in the paper, especially in how it tackles different stages in the research process. In our understanding, each tool adheres with the principles of open science. As such, we included the following paragraph in section IV: 

 “Huebner et al. (2021) introduced the TREQ approach, a set of four tools to reduce questionable research practices. These tools include preregistering analysis plans, reporting guidelines, sharing preprints of papers, and making data and code open. Each tool promotes openness at different stages of the research process and allows researchers to prevent issues like data manipulation, insufficient details of methods, and lack of reproducibility, among others.” 

**Comment 3:**

 The discussion of the publishing industry revenue structure could be given more depth. There have been quite a lot of changes, see e.g. here for a discussion on Plan S ( https://www.nature.com/articles/d41586-021-00883-6); there are different levels of open access (Gold vs. Green) etc. Also, I cannot quite follow the argument how creating different streams of revenue would aid open access. Many journals already are open access or have an open access option (even subscription journals). Is the idea here to replace the subscription model with an open access model through payment for individual services instead of a university subscribing to a journal / publisher? Could there be unintended consequences especially for more junior scholars / those not holding large grants? 

**Author Response:**

 We thank the reviewer for this comment. A similar point was brought forward by another reviewer. The analysis of the decoupling argument in the first version of the paper was misdirected. The paragraph has been re-written to better convey the original message put up by the referenced papers. We hope to have made the motivation and the argument clearer. 

**Comment 4:**

 About preprints being potentially problematic because of the lack of oversight, this is a fair point – but they bring many benefits (e.g. earlier and wider access to scientific findings; possibility to include not yet published work in grant applications by linking to a preprint) and it might be an option to simply shape a mindset that preprints come with a ‘health warning’. E.g. preprint servers could overlay all preprints with a clear message saying “not peer-reviewed, no assessment of validity and reliability done” and the onus would be on the reader to interpret them as preliminary. 

**Author Response:**

 We agree with the reviewer and incorporated part of the text of the comment in the updated version of the paper.

# Reviewer report: Reviewer response for version 1

**DOI:** 10.21956/openreseurope.16970.r31993

**Published:** 2023-07-19

**Authors:** Sacha Hodencq

**Affiliations:** 1 University Grenoble Alpes, Saint-Martin-d'Hères, Auvergne-Rhône-Alpes, France

The Open letter “Open science and ethics in energy research” introduces the context of open science in current research practices. After defining open science itself, it discusses its interests and limits, in particular in the energy research field.

 The letter provides an overview of current work and discussion on the topic, with a good number of references. It underlines the interests of open practices in the energy field, and the necessary distinction between openness and quality. It includes interesting conclusions on the need for open science despite its limitations, and the paradigm shift it necessitates.

 I believe the letter could still be improved. Generally speaking, by being more explicit about whether the subject is open science or its sub-components (in particular differentiating open science from open access) in order to avoid confusion, based on more recent references; and by clarifying the ethics mentioned in the title.

 This review generally follows the sequence of the letter.

 In the **section II.,** The definition of open science from Michael Nielsen that is provided seems indeed well established. A recent and comprehensive reference that could also be included is the UNESCO recommendation on Open Science ^1^ that explicitly encompasses various movements and practices in the Open Science definition, contrary to the following definition from the Guide to Open Science that focuses on Open access and, to a certain extent, on open data and open source software, or to the definition from Kraker et al. 2011 ^2^ (by the way, if this reference is kept, please quote the Kraker et al. 2011 article rather than the openscienceasap website that is not available in English). I believe including the UNESCO open science definition as well as recommendations could enrich the letter contents. This would for instance enable to include open hardware in addition to open source software in the 4 ^th^ paragraph of the section II., and give referenced definition of those concepts. The explicit mention of the 4 Mertonian values does not seem essential here, as their link with open science is not developed in the letter nor in the references from what I read (the link with universalism and communism seems intuitive, it is less the case with the other two), and these Mertonian values are not reused later in the letter. I would also point out that Mertonian values have been criticised as for their idealised view of the way research is carried out, but I understand this is out of the scope of this letter ^4^. An additional interesting reference in my view in the Open Science Training Handbook from the European program FOSTER ^3^, that was first released in 2018, and includes good definitions and details, for instance to support the open access and quality insurance paragraphs in section II.

 In **section III**., the first paragraphs explore ethics as the prevention from bad research practices. Energy systems are complex socio-technical systems with great environmental and social impacts, as mentioned in the introduction. So the energy research field call for both research ethics and integrity. In my view, the article focuses on integrity rather than ethics generally speaking. So my advice would be whether to expand on ethical aspects of energy research; or more realistically, to define more clearly the scope of ethical exploration from the title and introduction, as otherwise, one could expect deeper ethical development of open science in energy research. In the 4 ^th^ paragraph of section III.A., I do not fully understand why the functions decoupling is conditional: from my understanding, it sounds like the Golden open access model ^4^ that more and more journals are using (not the majority though). Moreover, I feel it should be made more explicit where the subject is open science generally speaking (as it is well done in the paragraph right before section III.A), and when the subject is open access. For instance in the last two paragraphs of section III., I feel that what is discussed is open access scientific publication, and not open science generally speaking.

 In the **section IV**., the 3 ^rd^ paragraph about terminologies could mention the Open Energy Ontology project ^5^, or generally speaking articles exploring the questions of ontology in energy modelling such as the one from Pritoni et al. ^6^. The 4 ^th^ paragraph of this section could mention the coalitionS that has achieved big steps in the open access publication of result from research funded by public grants ^7^. The 5 ^th^ paragraph could mention papers that specifically explored the benefits of open science in energy research, such as the ones from Morrison, Pfenninger et al., Bazilian et al. or. ^8^^,^^9^^,^^10^. For what it’s worth, I tried to synthetise these interests in ^11^. Then the checklist from Cao et al. ^12^ could be mentioned in the 7 ^th^ paragraph regarding the guidelines. In the 9 ^th^ paragraph, is the number of projects funded in the energy field known? If so, please share it here. In the 10 ^th^ paragraph, a very representative example of this is the International Energy Agency, see Ritchie and Roser ^13^. The last paragraph of this section could also mention the teaching materials from OSeMOSYS ^14^.

 In the **Discussion, **paragraph 4: Morrison ^8^ argues that the (time) resources consumed for openness is often outweighed by the external contributions, this might be worth mentioning it.

 Regarding the form of the letter:

- end of third paragraph of the *Introduction section*: “also” should be put before “be”
- section II., 5 ^th ^paragraph: rather than “other researchers are [..] incentivised”, I would say “other researchers are [..] offered the possibility”, as, in my view, they are not incentivised.
- There is a section III.A but no section III.B
- Section IV., 2 ^nd^ paragraph: I would say “in particular” rather than “however”, as to me, Pfenninger et al. go in the same direction as what was discussed beforehand.
- Section IV., 7 ^th^ paragraph: As mentioned on the Python website ^15^, I would present the programming languages as an “open-source” rather than “free access”.
- Section IV., 8 ^th^ paragraph: I would talk about “black-box models” rather than “black-boxes”, and then talk about open resources rather than “open access resources”. A good reference here is Pfenninger et al. ^8^.
- Section IV, in terms of flow of thoughts, I feel the paragraphs 11 and 12 could be brought closer to the 5 ^th^, as they seem to develop the interests of open science in the energy field.

*Disclaimer:* this review represents my current view on the topic, and can obviously be constructively criticised as well.

Where applicable, are recommendations and next steps explained clearly for others to follow? (Please consider whether others in the research community would be able to implement guidelines or recommendations and/or constructively engage in the debate)

Yes

Does the article adequately reference differing views and opinions?

Partly

Are all factual statements correct, and are statements and arguments made adequately supported by citations?

Partly

Is the rationale for the Open Letter provided in sufficient detail? (Please consider whether existing challenges in the field are outlined clearly and whether the purpose of the letter is explained)

Partly

Is the Open Letter written in accessible language? (Please consider whether all subject-specific terms, concepts and abbreviations are explained)

Yes

Reviewer Expertise:

My research mainly focuses on open science and energy modelling. I am a member of the French committee for Open Science.

I confirm that I have read this submission and believe that I have an appropriate level of expertise to confirm that it is of an acceptable scientific standard, however I have significant reservations, as outlined above.

## References

[1] UNESCO Recommendation on Open Science. *UNESCO Digital Library* .2021; Reference source

[2] : The case for an open science in technology enhanced learning. *International Journal of Technology Enhanced Learning* .2011;3(6) : 10.1504/IJTEL.2011.045454 10.1504/IJTEL.2011.045454

[3] : Open Science Training Handbook.2018; Reference source

[4] : An Epistemology for Democratic Citizen Science.2022; 10.31219/osf.io/j62sb 10.31219/osf.io/j62sb PMC1064646538026019

[5] : Introducing the Open Energy Ontology: Enhancing data interpretation and interfacing in energy systems analysis. *Energy and AI* .2021;5: 10.1016/j.egyai.2021.100074 10.1016/j.egyai.2021.100074

[6] : Metadata Schemas and Ontologies for Building Energy Applications: A Critical Review and Use Case Analysis. *Energies* .2021;14(7) : 10.3390/en14072024 10.3390/en14072024

[7] : Reference source

[8] : Energy system modeling: Public transparency, scientific reproducibility, and open development. *Energy Strategy Reviews* .2018;20: 10.1016/j.esr.2017.12.010 49-63 10.1016/j.esr.2017.12.010

[9] : Opening the black box of energy modelling: Strategies and lessons learned. *Energy Strategy Reviews* .2018;19: 10.1016/j.esr.2017.12.002 63-71 10.1016/j.esr.2017.12.002

[10] : Open source software and crowdsourcing for energy analysis. *Energy Policy* .2012;49: 10.1016/j.enpol.2012.06.032 149-153 10.1016/j.enpol.2012.06.032

[11] : Open and Reproducible Use Cases for Energy (ORUCE) methodology in systems design and operation: a dwelling photovoltaic self-consumption example. *In Building Simulation. Bruges, Belgium.* .2021; Reference source

[12] : Raising awareness in model-based energy scenario studies—a transparency checklist. *Energy, Sustainability and Society* .2016;6(1) : 10.1186/s13705-016-0090-z 10.1186/s13705-016-0090-z

[13] Reference source

[14] Reference source

[15] Reference source

# Response

**Published:** 2025-01-14

**Authors:** Raquel Alonso Pedrero

**Affiliations:** Norwegian University of Science and Technology, Norway

Thanks for your constructive feedback and interesting question raised. General Comments: 

 The Open letter “Open science and ethics in energy research” introduces the context of open science in current research practices. After defining open science itself, it discusses its interests and limits, in particular in the energy research field. The letter provides an overview of current work and discussion on the topic, with a good number of references. It underlines the interests of open practices in the energy field, and the necessary distinction between openness and quality. It includes interesting conclusions on the need for open science despite its limitations, and the paradigm shift it necessitates.    I believe the letter could still be improved. Generally speaking, by being more explicit about whether the subject is open science or its sub-components (in particular differentiating open science from open access) in order to avoid confusion, based on more recent references; and by clarifying the ethics mentioned in the title. 

**Response:**

 Thank you for your comments and your feedback. We have addressed all the concerns in the comments below. In the new manuscript, we aimed to be more explicit and clear about the ideas we wanted to convey and be more cautious with the wording. We remove the ethics mentioned in the title and focus on presenting open science and its benefits, barriers and integration in energy research. 

 Comment 1: 

 In the section II., The definition of open science from Michael Nielsen that is provided seems indeed well established. A recent and comprehensive reference that could also be included is the UNESCO recommendation on Open Science 1 that explicitly encompasses various movements and practices in the Open Science definition, contrary to the following definition from the Guide to Open Science that focuses on Open access and, to a certain extent, on open data and open source software, or to the definition from Kraker et al. 2011 2 (by the way, if this reference is kept, please quote the Kraker et al. 2011 article rather than the openscienceasap website that is not available in English). I believe including the UNESCO open science definition as well as recommendations could enrich the letter contents. This would for instance enable to include open hardware in addition to open source software in the 4th paragraph of the section II., and give referenced definition of those concepts. 

**Response:**

 We really appreciate the suggestions and insights provided. In the new manuscript, we kept Michael Nielsen's and the Guide to Open Science's definitions, given their well-established status. Then, we clarified that open science integrates more principles based on Kraker et al. (2011) and UNESCO (2021). Also, following the UNESCO guidelines and your suggestion, we added open hardware as one of the key principles. 

 Comment 2: 

 The explicit mention of the 4 Mertonian values does not seem essential here, as their link with open science is not developed in the letter nor in the references from what I read (the link with universalism and communism seems intuitive, it is less the case with the other two), and these Mertonian values are not reused later in the letter. I would also point out that Mertonian values have been criticised as for their idealised view of the way research is carried out, but I understand this is out of the scope of this letter 4. 

**Response:**

 We share your concern that linking the Mertonian values with open science in the paper is separate from the rest of the paper’s content. Given that the topic is open science in energy research, we eliminated the word ethics from the title and removed the text referring to the Mertonian values from Section I. Still, we kept this intuitive connection with universalism and communism in the introduction as a way of emphasizing the alignment between open science and the ethos in science and as a starting point for the paper.  

 Comment 3: 

 An additional interesting reference in my view in the Open Science Training Handbook from the European program FOSTER 3, that was first released in 2018, and includes good definitions and details, for instance to support the open access and quality insurance paragraphs in section II. 

**Response:**

 This was a valuable comment. In the new manuscript, the structure in section I is divided into three new subsections, one addressing its integration (C. Applying open science). In this section, the Open Science Training Handbook, in combination with the FAIR principles, are presented as guidelines or frameworks to support the adoption and practice of open science. 

 Comment 4: 

 In section III., the first paragraphs explore ethics as the prevention from bad research practices. Energy systems are complex socio-technical systems with great environmental and social impacts, as mentioned in the introduction. So the energy research field call for both research ethics and integrity. In my view, the article focuses on integrity rather than ethics generally speaking. So my advice would be whether to expand on ethical aspects of energy research; or more realistically, to define more clearly the scope of ethical exploration from the title and introduction, as otherwise, one could expect deeper ethical development of open science in energy research. 

**Response:**

 We appreciate the reviewer highlighting the distinction between ethics and integrity in research. The old manuscript failed to address clear ethical aspects such as environmental impact, social justice, public engagement, etc. Instead, it was more focused on the conduct and process of research. One can argue that open science tackles the ethical concern of transparency, but the title and the introduction still led to the expectation of a more detailed discussion on ethics. The new manuscript was rewritten focusing on research integrity through open science and its application in energy research. As such, the title was modified by removing the term ethics. 

 Comment 5:  

 In the 4th paragraph of section III.A., I do not fully understand why the functions decoupling is conditional: from my understanding, it sounds like the Golden open access model 4 that more and more journals are using (not the majority though).  Moreover, I feel it should be made more explicit where the subject is open science generally speaking (as it is well done in the paragraph right before section III.A), and when the subject is open access. For instance in the last two paragraphs of section III., I feel that what is discussed is open access scientific publication, and not open science generally speaking.  

**Response: ** We thank the reviewer for this comment. A similar point was brought forward by another reviewer. The analysis of the decoupling argument in the first version of the paper was misdirected. The paragraph has been re-written to better convey the original message put up by the referenced papers. We hope to have made the motivation and the argument clearer. 

 Comment 6: 

 In the section IV., the 3rd paragraph about terminologies could mention the Open Energy Ontology project 5, or generally speaking articles exploring the questions of ontology in energy modelling such as the one from Pritoni et al. 6. 

**Response:**  We thank the reviewer for this comment. Both references were included in section IV, subsection A. 

 Comment 7: 

 The 4th paragraph of this section (section IV) could mention the coalitionS that has achieved big steps in the open access publication of result from research funded by public grants 7. **Response:**  Thank you for the suggestion. CoalitionS and Plan S focus on open access across all scientific disciplines, not just energy research. Therefore, we believe it is appropriate to mention them in Section II C. Applying open science, where we discuss the application of open science in a wider context. 

 Comment 8: 

 The 5th paragraph could mention papers that specifically explored the benefits of open science in energy research, such as the ones from Morrison, Pfenninger et al., Bazilian et al. or. 8,9,10. For what it’s worth, I tried to synthetise these interests in 11. 

**Response:**  We thank the reviewer for those most important sources. We make use of those references mostly in section IV.  

 Comment 9: 

- Then the checklist from Cao et al. 12 could be mentioned in the 7th paragraph regarding the guidelines.  

- In the 9th paragraph, is the number of projects funded in the energy field known? If so, please share it here. 

- In the 10th paragraph, a very representative example of this is the International Energy Agency, see Ritchie and Roser 13. 

- The last paragraph of this section could also mention the teaching materials from OSeMOSYS 14. 

- In the Discussion, paragraph 4: Morrison 8 argues that the (time) resources consumed for openness is often outweighed by the external contributions, this might be worth mentioning it. 

**Response: **

- We thank the reviewer for the reference suggestion, In the new version of the manuscript, the work of Cao et al. (2016) was mentioned alongside other relevant papers in section IV, subsection C. 

- Based on this and other comments, we believe that the section comparing the adherence to open science between disciplines was not sufficiently detailed and was out of the scope of the paper. Thus, it is no longer part of the paper. 

- We included this reference in section IV, subsection IV, citing alongside renewables.ninja. 

- The teaching materials from OSeMOSYS and the link to its webpage are included in the new manuscript (last paragraph Section IV). 

- Thanks for raising this point, we found it a valid limitation to open source. We included it in Section IV B. 

Comment 10:  Regarding the form of the letter: 

- End of third paragraph of the Introduction section: “also” should be put before “be”. 

- Section II., 5th paragraph: rather than “other researchers are [..] incentivised”, I would say “other researchers are [..] offered the possibility”, as, in my view, they are not incentivised.   

- There is a section III.A but no section III.B   

- Section IV., 2nd paragraph: I would say “in particular” rather than “however”, as to me, Pfenninger et al. go in the same direction as what was discussed beforehand. 

- Section IV., 7th paragraph: As mentioned on the Python website 15, I would present the programming languages as an “open-source” rather than “free access”. 

- Section IV., 8th paragraph: I would talk about “black-box models” rather than “black-boxes”, and then talk about open resources rather than “open access resources”. A good reference here is Pfenninger et al. 8. 

- Section IV, in terms of flow of thoughts, I feel the paragraphs 11 and 12 could be brought closer to the 5th, as they seem to develop the interests of open science in the energy field. 

Disclaimer: this review represents my current view on the topic, and can obviously be constructively criticised as well. 

**Response: **

- The referred sentence is no longer part of the new manuscript. 

- We agree “incentivised” was not correct in this context. Nonetheless, we took out this sentence from the manuscript. 

- The new manuscript divides Section III into five subsections, solving the issue mentioned. 

- True. The sentence was rewritten as:  
  “Transparency on models and assumptions not only addresses mistrust from society but also benefits the development of the research domain by enhancing feedback and enabling the correction of errors. Pfenninger et al. [31] identify other three beneficial outcomes of adhering to open science in energy research” 

- Thanks for the suggestion, the word was modified to keep consistency throughout the text. 

- We now refer only to “black-box models” and “open resources”. 

- Thanks for the suggestion. We felt that Section IV needed to be reorganized based on specific ideas. We hope the new version presents a more ordered flow of thought. 

# Reviewer report: Reviewer response for version 1

**DOI:** 10.21956/openreseurope.16970.r31997

**Published:** 2023-07-19

**Authors:** Rosie Robison, Ami Crowther

**Affiliations:** 1 Anglia Ruskin University, Chelmsford, England, UK

This is an interesting paper which draws out and reflects open science within the context of energy research. Within the paper, focus is placed upon the ethical dimensions of open science, although we feel this key message should be made more prominent in the paper’s narrative before approval.

**General Points**

 Overall, the paper is well written but greater effort could be made to ensure the overarching narrative is clear and the central points are emphasised. The use of more ‘signposting’ would support the development of this narrative and ensure that the reader is on the intended journey. Signposting would include summarising the points that will be covered in each section, and linking between the different sections (showing how they relate to each other and in combination contribute towards the narrative). For example, having a more structured approach in the discussion of open science through outlining the broad themes that will be discussed.

 The link to ethics is an interesting angle and one that could be made more explicit within the main body of the paper, rather than being mentioned in only the introduction and conclusion of the paper.

 It feels like some of the points would benefit from additional references to support the points being made, as well as showing how this paper fits within current knowledge and practices.  

 At times, the authors introduce terms but don’t really define them – for example, black-box models. Including short definitions of these terms, within the context of the paper, could help with reader comprehension and increase the impact of the points made.

 To tighten the focus of the paper (on the ethics of open science in relation to energy) there is the opportunity to refine the literature discussed and focus on ideas that are of relevance to the overarching focus of the paper. For example, when discussing the prevention of bad research practices could this be linked back to energy or ethics in some way?

 When listing multiple points, we feel the use of numbering would really improve the readability and clearly show the points that are being made. For example, when listing the functions of mainstream journals (page 4-5).

**Introduction**

 Is there a way of mentioning ethics within this opening section?

 Could additional references be included to provide the context/set up the need for this paper?

 The stated aim of the paper is to analyse the adoption of open science and analyse its integration in the energy research domain – how is this being done i.e. through what methodology? Is it based upon a literature review or the researcher’s experiences?

**Open Science: Definition**

 When introducing the six principles of open science, signposting could be included in the paper along the lines of ‘each of these principles will now be briefly introduced’.

 At the end of the section, it would be useful to have a linking section of how this is relevant for the rest of the paper or link back to the idea of energy research.

**Open science for prevention of bad research practices**

 Could the term ‘bad research practices’ be made more specific? This is slightly informal language at the moment.

 This section could be set up in a clearer way – briefly summarising the main argument/s that are going to be discussed would be useful for the reader and would bring a flow to the paper.

 The US Office of Research Integrity quote could perhaps be shortened.

 The use of Feigenbaum and Levy’s drawbacks of sharing scientific resources is a good way to set up the paragraph, perhaps an additional sentence saying ‘these will now be discussed in more detail’ would help readers, and a short discussion of the ‘interference with future work’.

 This section feels like a list of separate thoughts that are just put in a sequence one after the other, it would be nice if some of the points could be developed a bit further (and perhaps the number of points being made could be reduced to enable this and ensure you keep within the word count).

 The mention of altmetrics is an interesting one, particularly as this provides a means for addressing the risk of bad research provided. It feels like greater detail could be provided on what is covered by altmetics and why they themselves are not an ‘incorrect’ incentive.

**Open science and energy research**

 We feel the word ‘however’ may not be needed before Pfenniger on page 5 as this suggests that this reference indicates that there are not positive outcomes, whereas the Pfenniger reference is actually highlighting four prevalent outcomes.

 It would be nice to have an elaboration on how the homogenisation of metadata contributes towards the common understanding being established and in what way is data homogenised through open science.

 We are not sure the relevance of the analogy related to optimisation tools – how does this relate to the overarching aim/narrative of the paper?

 Perhaps an area to explore is why there may be variations between disciplines in terms of uploads to the Open Data Portal – what understandings, approaches, methods, schools of thought exist within these disciplines contributes to them uploading (or not uploading to the Open Data Portal).

 It would be nice to more clearly set out the key messages that you’re wanting to convey when presenting the different examples in this section, how do they come together and what do they say? Some sentences at the end of the paragraphs summarising responses to these questions would help guide the author through the section.

**Discussion**

 The opening paragraph of this section is really effective and links directly to the aim outlined in the introduction. The points outlined in this paragraph could be better emphasised throughout the main body of the text – these summary phrases could be used within the signposting.

 As mentioned in the ‘General Points’ section of our review, there is the opportunity to have a more structured approach to the discussion of open science. A more structured approach may be facilitated by reducing the breadth of topics covered and go into greater detail on a few key points. In the current configuration, certain points could be expanded further, as the reader is left thinking ‘so what?’. These key points could then include greater reference to the ethics framing adopted within the paper.

Where applicable, are recommendations and next steps explained clearly for others to follow? (Please consider whether others in the research community would be able to implement guidelines or recommendations and/or constructively engage in the debate)

Partly

Does the article adequately reference differing views and opinions?

Partly

Are all factual statements correct, and are statements and arguments made adequately supported by citations?

Partly

Is the rationale for the Open Letter provided in sufficient detail? (Please consider whether existing challenges in the field are outlined clearly and whether the purpose of the letter is explained)

Partly

Is the Open Letter written in accessible language? (Please consider whether all subject-specific terms, concepts and abbreviations are explained)

Partly

Reviewer Expertise:

Energy-related Social Sciences and Humanities

We confirm that we have read this submission and believe that we have an appropriate level of expertise to state that we do not consider it to be of an acceptable scientific standard, for reasons outlined above.

# Response

**Published:** 2025-01-14

**Authors:** Raquel Alonso Pedrero

**Affiliations:** Norwegian University of Science and Technology, Norway

Thanks for your comments, and sorry for our late response. We hope the new manuscript has a more straightforward message and that presents the discussion with a better narrative. General Comments: 

 This is an interesting paper which draws out and reflects open science within the context of energy research. Within the paper, focus is placed upon the ethical dimensions of open science, although we feel this key message should be made more prominent in the paper’s narrative before approval.  Response: 

 Thank you for the feedback. We understand the concern about the key message, and we hope that you find the new manuscript much clearer and coherent. We addressed all of your comments below. 

**Comment 1:**

 Overall, the paper is well written but greater effort could be made to ensure the overarching narrative is clear and the central points are emphasised. The use of more ‘signposting’ would support the development of this narrative and ensure that the reader is on the intended journey. Signposting would include summarising the points that will be covered in each section, and linking between the different sections (showing how they relate to each other and in combination contribute towards the narrative). For example, having a more structured approach in the discussion of open science through outlining the broad themes that will be discussed. 

**Response:**

 We appreciate your feedback. We agreed that the old manuscript lacks a proper flow in the narrative which could make the reading challenging. We introduced signposting in all the sections of the new manuscript by i) initiating/finishing the sections and subsections with a reference to their following, ii) referring more often to concepts stated in previous sections to improve the coherence of the paper, iii) including bullet points and iv) incorporating subsections that connect with more broad themes. An example of this last point is the division of Section IV. Now, it is divided into A. Benefits of Open Science in Energy Research, B. Barriers and Limitations of Open Science in Energy Research and C. Integration of Open Science in Energy Research. These subsections are named the same as previous ones but with a focus on Energy Research. In this way, the new manuscript states a clear outline as suggested. 

**Comment 2:**

 The link to ethics is an interesting angle and one that could be made more explicit within the main body of the paper, rather than being mentioned in only the introduction and conclusion of the paper. 

**Response:**

 Thank you for appreciating the link to ethics. Based on the different reviewers and the rest of the paper, we decided to reduce the relevance of ethics and focus more on the integrity/application of open science. This is also stated by modifying the title from “Open science and ethics in energy research” to “Open science in energy research”. However, we also believe that the link with ethics is still worth mentioning, so it is included in the introduction of the new manuscript.  

**Comment 3:**

 It feels like some of the points would benefit from additional references to support the points being made, as well as showing how this paper fits within current knowledge and practices. **Response:** 

 While the previous version of the article had 34 references, this number has increased to 45 in the current format. This accounts for the inclusion of important references suggested by the reviewers and new references added by the authors to support the points being made. **Comment 4:**

 At times, the authors introduce terms but don’t really define them – for example, black-box models. Including short definitions of these terms, within the context of the paper, could help with reader comprehension and increase the impact of the points made.  

**Response:**

 Thank you for pointing out the need for clearer words. We have addressed this by including a simple definition of black box models in a footnote to enhance reader comprehension. Additionally, we reviewed the manuscript and ensure that other technical terms were clarified to improve the accessibility of the paper. 

**Comment 4:**

 To tighten the focus of the paper (on the ethics of open science in relation to energy) there is the opportunity to refine the literature discussed and focus on ideas that are of relevance to the overarching focus of the paper. For example, when discussing the prevention of bad research practices could this be linked back to energy or ethics in some way? 

**Response:**

 We appreciate your feedback. Also, other reviewers noticed a lack of “connection” between parts of the paper. As such, the paper is structured such that the first part (Sections II and III) introduces open science in general, and the second part (Sections IV and V) dives into its impact, barriers and application in energy research. In the second part, the terminology from the first section is used regularly in the new manuscript to, as you suggested, discuss the links between ideas. We hope we have addressed this concern properly, as we believe it is key to the paper's quality. 

**Comment 5:**

 When listing multiple points, we feel the use of numbering would really improve the readability and clearly show the points that are being made. For example, when listing the functions of mainstream journals (page 4-5).  

**Response:**

 We agree. As such, we integrated numbering and bullet points to make a clearer statement of lists or points. For instance, the key principles of open science in Section II are now presented in numbered bullet points, significantly improving readability and clarity. 

**Comment 6**: 

 Introduction 

- Is there a way of mentioning ethics within this opening section?   

- Could additional references be included to provide the context/set up the need for this paper?   

- The stated aim of the paper is to analyse the adoption of open science and analyse its integration in the energy research domain – how is this being done i.e. through what methodology? Is it based on a literature review or the researcher’s experiences? 

**Response:** 

- As mentioned in a previous response, the paper has been redefined not to be specific to ethics. Still, it is mentioned in Section I as a starting point for introducing open science as suggested. 

- The paper includes new references to set up a better context and back up the ideas presented. However, we wanted to point out that as an open letter, we also include ideas from our own experience that may not have been so clearly stated in previous references. 

- Thank you for pointing out the lack of clarity on implementation. The new manuscript dives more into this. We introduce the frameworks of FAIR and the Open Science Training Handbook and use them in the rest of the paper. Also, we introduce the subsections: Applying open science (Section II) and Integration of Open Science in Energy Research to discuss how open science can be integrated and what is the current status of integration in energy research. 

**Comment 7:**

 Open Science: Definition   

- When introducing the six principles of open science, signposting could be included in the paper along the lines of ‘each of these principles will now be briefly introduced’.   

- At the end of the section, it would be useful to have a linking section of how this is relevant for the rest of the paper or link back to the idea of energy research.  

**Response: **

- The principles of open science are now presented in numbered bullet points. 

- We appreciate this suggestion. We agreed that there is a lack of linkage between section II and energy research in the old manuscript. This also extends to section III. After considering the different options to tackle it, we believe that the more straightforward way to tackle this concern is to start Section IV as follows: 
  “Learning from the definitions, practices, benefits, and shortcomings of open science presented in the previous sections, the following focuses on the link these concepts to the particular domain of energy research.” 

 

 Also, the entire text is linked by using the same terminology across sections. We hope these solutions, along with the structure change, help identify connections between ideas. **Comment 8:**

 Open science for prevention of bad research practices   

- Could the term ‘bad research practices’ be made more specific? This is slightly informal language at the moment. 

- This section could be set up in a clearer way – briefly summarising the main argument/s that are going to be discussed would be useful for the reader and would bring a flow to the paper. 

- The US Office of Research Integrity quote could perhaps be shortened. 

- The use of Feigenbaum and Levy’s drawbacks of sharing scientific resources is a good way to set up the paragraph, perhaps an additional sentence saying ‘these will now be discussed in more detail’ would help readers, and a short discussion of the ‘interference with future work’. 

- This section feels like a list of separate thoughts that are just put in a sequence one after the other, it would be nice if some of the points could be developed a bit further (and perhaps the number of points being made could be reduced to enable this and ensure you keep within the word count). 

- The mention of altmetrics is an interesting one, particularly as this provides a means for addressing the risk of bad research provided. It feels like greater detail could be provided on what is covered by altmetics and why they themselves are not an ‘incorrect’ incentive.  

**Response: **

- Fixed by referring to inappropriate practices 

- The section is now divided into subsections, and each idea is briefly introduced in the title to facilitate identification of the arguments. 

- As suggested, the definition was reduced as details were not discussed further. 

- Thaks for noticing this issue in the readability. The discussion on issues for sharing scientific resources is now presented in two distinct subsections: 1) Additional Effort and Time Consumption and 2) Impact on Future Work and Competitiveness. This division facilitates the identification of the arguments and discussions. 

- We hope the new structure and the rewritten discussions ease the reading flow. 

- We thank the reviewer for the suggestion to deepen the understanding of Altmetrics. We expanded the explanation with an example, as we feel that a deeper dive into Altmetrics is beyond the scope of the text. 

**Comment 9:**

 Open science and energy research   

- We feel the word ‘however’ may not be needed before Pfenniger on page 5 as this suggests that this reference indicates that there are not positive outcomes, whereas the Pfenniger reference is actually highlighting four prevalent outcomes.  

- It would be nice to have an elaboration on how the homogenisation of metadata contributes towards the common understanding being established and in what way is data homogenised through open science.    

- We are not sure the relevance of the analogy related to optimisation tools – how does this relate to the overarching aim/narrative of the paper?   

- Perhaps an area to explore is why there may be variations between disciplines in terms of uploads to the Open Data Portal – what understandings, approaches, methods, schools of thought exist within these disciplines contributes to them uploading (or not uploading to the Open Data Portal).   

- It would be nice to more clearly set out the key messages that you’re wanting to convey when presenting the different examples in this section, how do they come together and what do they say? Some sentences at the end of the paragraphs summarising responses to these questions would help guide the author through the section. 

**Response: **

- No longer applies 

- Based on the research from Wierling et al., it has been shown that energy data and metadata are usually decentralized and not accessible to energy researchers, such that standards and nomenclature are not homogenised and standardised. Adopting centralised and open access platforms could enforce uniformity in how data and metadata are structured and shared and provide a common repository to eliminate double efforts. 

- We have removed the analogy to optimisation in the effort to make the paper more general and accessible.  

- Thank you for your suggestion. We also believe that this is an interesting area of research and as such it was mentioned in the discussion section. 

- Thanks for your feedback. After revision, we also perceived a need for coherence and clarity. The entire section was rewritten with this in mind. The first noticeable change that could guide the reader better on the key messages is the division into three subsections. Each key idea is presented in a paragraph. For instance, in Section A, we start by linking it with the previous paragraph and introducing the benefits pointed out by Pfenninger. Each of these benefits is discussed in the following paragraphs. Finally, the additional benefit of homogenisation of metadata is clearly stated and discussed.  

**Comment 10:**

 Discussion 

- The opening paragraph of this section is really effective and links directly to the aim outlined in the introduction. The points outlined in this paragraph could be better emphasised throughout the main body of the text – these summary phrases could be used within the signposting.   

- As mentioned in the ‘General Points’ section of our review, there is the opportunity to have a more structured approach to the discussion of open science. A more structured approach may be facilitated by reducing the breadth of topics covered and go into greater detail on a few key points. In the current configuration, certain points could be expanded further, as the reader is left thinking ‘so what?’. These key points could then include greater reference to the ethics framing adopted within the paper. 

**Response:**  Thank you for this suggestion. We believe that the current version of the discussion is improved due to important points flagged by the reviewers. Also, we restructured it such that we focused on the summarising the insights from the rest of the article and to showcase that open science has both advantages and barriers.

# Reviewer report: Reviewer response for version 1

**DOI:** 10.21956/openreseurope.16970.r31995

**Published:** 2023-07-14

**Authors:** Stephan Ferenz, Thomas Wolgast

**Affiliations:** 1 University of Oldenburg, Oldenburg, Germany

**General**

 The article gives an overview of the different aspects of Open Science and attempts to show the current state of Open Science in Energy Research.

 The introduction nicely outlines the latest developments to Open Science and its relevance. It motivates why the state of Open Science in energy research is an important topic and relevant to analyze.

 The second section introduces the concept of Open Science. While this introduction gives a good overview of six parts of Open Science (Open Access, Open Methodology, Open Data, Open Source, Open Review, Open Educational Resources) it would profit a lot when also introducing the concept of FAIR (doi.org/10.1038/sdata.2016.18 ^1^) in respect to research data or research software (doi.org/10.3233/DS-190026 ^2^) and connecting it to Open Science. Also, this section is rather general and should be more specific.

 The third section also addresses the general topic of Open Science and outlines how Open Science can improve research. While this is an important topic, its connection to energy research can be improved. Also, this section can be improved by reusing the categorization of the different aspects of Open Science from section II. Since Open Science in energy research is the main scope of the paper, we would recommend to shorten this section.

 Finally, the fourth section analyzes Open Science in energy research. As in section III, this section can be improved by reusing the categorization of Open Science from section II. This section lists multiple examples that need to be connected more to a common theme. While most aspects have a connection to energy research, same are only vague connect, e.g. the Open Courseware in the last paragraph of the section. In some paragraphs of this section, the paper tries to differentiate between different areas of energy research (more industry related vs. more policy related) and their different levels and reasons for and against open science. This is an important aspect that should be analyzed with more effort and with more examples from the different areas of energy research. Furthermore, it should be discussed how the security aspect of research on the critical infrastructure plays a role here. Since this section is the main section of the paper, it would profit from dividing it into subsections. Also, this topic should be the longest part of the paper.

 The last section of the paper discusses the different aspects of Open Science and outlines advantages and disadvantages. This part would profit from showing the connection of the different aspects to energy research.

 We think the paper would also profit from an outlook section which could point out what additional research is needed to get a better overview of the state of Open Science in energy research.

**Major Issues**

1. In the context of open source, the term and concept of research software is important and should be integrated (e.g. doi.org/10.3233/DS-190026 ^2^).
2. The third paragraph in section IV focuses on metadata. Here, a link to existing approaches for metadata in the energy domain would be valuable (e.g. doi.org/10.3390/en14206692 ^3^).
3. The paper includes one figure. Unfortunately, the figure is not reproducible. The paper should reference to the actual dataset of the figure.
4. p6, "The number of entries for the energy category is remarkably low compared to other research fields": Well, it's a far more specialized field. This data is almost worthless without the number of publications or researchers in the respective fields for reference.
5. Publicly open conference are listed in the paragraph focusing on open educational resources. Scientific conferences are no educational resources. Also, it remains debatable if conference with conference fees can be counted as open.
6. The citation style with hyperlinks and without further information makes the links unusable if printed. Also, some examples (e.g. OpenMod) are also missing links. The missing links should be added and all links should also marked in an additional way, e.g. footnotes.

 

**Minor Issues**

1. Section III has a subsection A but no subsection B. We would recommend to remove the subheading from this subsection.
2. The paper mainly uses the term energy research but sometimes also energy science. An uniform use of one term would improve the paper.
3. p4 "Allowing other researchers to reuse the data and methods [...] enhances the chance to detect bad research practices." -> Would the respective people use Open Science then? How does Open Science needs to be implemented to help with this problem?
4. p4/5, "barrier to implementing open access is related to the publishing industry revenue structure": Where exactly is the barrier here? The paragraph sounds like it's no problem at all.
5. p6: Is Energies a high-profile journal? MDPI is considered to be predatory by many researchers. (e.g. https://academic.oup.com/rev/article/30/3/405/6348133 ^4^)
6. p6, "Not pursuing openness...": This whole paragraph and the following one are difficult to understand.

Where applicable, are recommendations and next steps explained clearly for others to follow? (Please consider whether others in the research community would be able to implement guidelines or recommendations and/or constructively engage in the debate)

No

Does the article adequately reference differing views and opinions?

Partly

Are all factual statements correct, and are statements and arguments made adequately supported by citations?

Partly

Is the rationale for the Open Letter provided in sufficient detail? (Please consider whether existing challenges in the field are outlined clearly and whether the purpose of the letter is explained)

Yes

Is the Open Letter written in accessible language? (Please consider whether all subject-specific terms, concepts and abbreviations are explained)

Yes

Reviewer Expertise:

Stephan Ferenz: Research data management and Open Science in Energy Research. Thomas Wolgast: Power Engineer interested in Open Science.

We confirm that we have read this submission and believe that we have an appropriate level of expertise to state that we do not consider it to be of an acceptable scientific standard, for reasons outlined above.

## References

[1] : The FAIR Guiding Principles for scientific data management and stewardship. *Sci Data* .2016;3: 10.1038/sdata.2016.18 160018 10.1038/sdata.2016.18 26978244 PMC4792175

[2] : Towards FAIR principles for research software. *Data Science* .2020;3(1) : 10.3233/DS-190026 37-59 10.3233/DS-190026

[3] : FAIR Metadata Standards for Low Carbon Energy Research—A Review of Practices and How to Advance. *Energies* .2021;14(20) : 10.3390/en14206692 10.3390/en14206692

[4] : Journal citation reports and the definition of a predatory journal: The case of the Multidisciplinary Digital Publishing Institute (MDPI). *Research Evaluation* .2021;30(3) : 10.1093/reseval/rvab020 405-419a 10.1093/reseval/rvab020

# Response

**Published:** 2025-01-14

**Authors:** Raquel Alonso Pedrero

**Affiliations:** Norwegian University of Science and Technology, Norway

Thanks for your valuable review and sorry for the late response. We hope this new manuscript and our responses tackle your comments appropriately.

**General Comments: **The article gives an overview of the different aspects of Open Science and attempts to show the current state of Open Science in Energy Research. 

 The introduction nicely outlines the latest developments to Open Science and its relevance. It motivates why the state of Open Science in energy research is an important topic and relevant to analyze. 

**Response: **Thank you for the detailed and meaningful feedback. It has definitely helped with the quality of the paper. We addressed each of the comments independently from each other bellow. 

**Comment 1: **The second section introduces the concept of Open Science. While this introduction gives a good overview of six parts of Open Science (Open Access, Open Methodology, Open Data, Open Source, Open Review, Open Educational Resources) it would profit a lot when also introducing the concept of FAIR (doi.org/10.1038/sdata.2016.181) in respect to research data or research software (doi.org/10.3233/DS-1900262) and connecting it to Open Science. Also, this section is rather general and should be more specific. **Response: **Thank you for this suggestion. The FAIR concept is fundamental among researchers’ practices nowadays (e.g., research proposals), thus the articles benefited from introducing it. The new manuscript includes subsection C. Applying open science (in Section II) where we included information from the two sources suggested. Also, it was complemented by the introduction to The Open Science Training Handbook, which also provides a framework/guidelines for supporting researchers. 

**Comment 2: **The third section also addresses the general topic of Open Science and outlines how Open Science can improve research. While this is an important topic, its connection to energy research can be improved. 

**Response: **We appreciate your suggestion. We agree that the old manuscript did not link well enough the barriers and shortcomings of Open Science with energy research. As such, we explicitly mentioned the connection with the different barriers proposed by including subsection B. Barriers and limitations of Open Science in Energy Research in Section IV. We decided to keep Section III still open and general and specify the link with Energy Research in the following section. 

**Comment 3: **Also, this section can be improved by reusing the categorization of the different aspects of Open Science from section II. 

**Response: **Similarly to the response to Comment 2, Section III makes more explicit usage of the principles of Open Science. 

**Comment 4: **Since Open Science in energy research is the main scope of the paper, we would recommend to shorten this section (referring to Section II). 

**Response: **Thank you for the recommendation. Given some important points added (e.g., FAIR principles), and the feedback from the other reviewers, Section II was not shortened in the new manuscript. However, we understand the concern of the reviewer, so the section was rewritten to improve its flow and reduce redundant information. For example, we remove the link with the Mertonian values and focus on the specific definition of Open Science. Also, we include more signposting to support the narrative. We hope these modifications helps to address the reviewer’s concern. We believe that, despite its length, this section introduces section IV (main scope). 

**Comment 5: **Finally, the fourth section analyses Open Science in energy research. As in section III, this section can be improved by reusing the categorization of Open Science from section II. 

**Response: **We agree. Therefore, the fourth section in the new manuscript elaborates in detail how the principles of Open Science tackle specific issues in energy research.  The explicit mention of the principles is included throughout the entire section. Just to give an example, in the new manuscript we address the influence of lobbies and interest groups on quantitative research by emphasizing the principles of open methodology, open source, and open data: 

 “A notable example of mistrust stemming from a lack of transparent and accessible energy research is the concern that lobbies or interest groups could influence its results. In quantitative research, where the main tools used are mostly mathematical models, results are particularly sensitive to the data selection, allowing for potential pressure from interest groups to sway the interpretation and generation of results. To mitigate this mistrust, it is crucial to prioritise openness. This can be achieved by implementing open methodology (clear documentation of the methods), open source (providing access to the tools used), and open data (making data accessible). Such transparency is key to identifying assumptions, inaccuracies, and limitations in research and essential for recognising and addressing potential pressures from interested parties.” 

**Comment 6: **This section [Section IV] lists multiple examples that need to be connected more to a common theme. While most aspects have a connection to energy research, same are only vague connect, e.g. the Open Courseware in the last paragraph of the section. **Response: ** We thank the reviewer for the comment. We modified the section substantially to address this concern. In the new manuscript Section IV is divided into three subsections to be more direct on the topics discussed and how they relate to open science. Moreover, we added more detailed descriptions to clarify the connection to energy research in particular, even using our own experiences as examples. 

**Comment 7: **In some paragraphs of this section, the paper tries to differentiate between different areas of energy research (more industry related vs. more policy related) and their different levels and reasons for and against open science. This is an important aspect that should be analyzed with more effort and with more examples from the different areas of energy research. 

**Response: **We think you have a valid point. We have revised the section to better differentiate between industry and policy-related energy research. For instance, in the “Barriers and Limitations of Open Science in Energy Research”, we address the competitive and legal concerns in industry-driven research (e.g., proprietary models) versus policy-driven research that is often publicly funded. We also discuss, in section “Benefits of Open Science in Energy Research”, the heterogeneity between different areas of energy research and how centralized approaches of data sharing could enhance uniformity in how data and metadata are structured. We are confident that the new structure of the Section will clarify this concern by having a more coherent flow of ideas. 

**Comment 8: **Furthermore, it should be discussed how the security aspect of research on the critical infrastructure plays a role here.  

**Response: ** We agree that this is an important topic of research and we appreciated this comment. We included the following paragraph in section IV addressing the trade-off between openness and security: 

 “Another area that faces challenges adhering to open science is research on critical infrastructure and cybersecurity.  These fields are essential for securing key societal technologies, such as electricity and gas networks. As with the trade-off between innovation and restricted access to information, the risk of exposing sensitive security data and methods outweighs the benefits of making it public. In this case, the potential harm might be too great to justify complete openness.” 

**Comment 9: **Since this section is the main section of the paper, it would profit from dividing it into subsections. Also, this topic should be the longest part of the paper. 

**Response: **Thanks for the suggestion. The section is now divided into three subsections: A. Benefits of Open Science in Energy Research, B. Barriers and Limitations of Open Science in Energy Research and C. Integration of Open Science in Energy Research. These subsections were developed following the flow of the previous two sections. This suggestion helped to significantly improve the paper by stating a clearer connection with the rest of the paper (addressing previous comments). Also, we highlight the importance of the section by expressing and signposting the goal of the section at the beginning of section IV. 

**Comment 10: **The last section of the paper discusses the different aspects of Open Science and outlines advantages and disadvantages. This part would profit from showing the connection of the different aspects to energy research. 

**Response: **We agree that the previous version lacked a proper link between energy research and open science. Following this recommendation, we decided to use Section IV to present more explicitly the aspects of open science and energy research. 

**Comment 11: **We think the paper would also profit from an outlook section which could point out what additional research is needed to get a better overview of the state of Open Science in energy research. 

**Response: **Thank you, we found this comment very meaningful to define better the scope of the paper. After the review we realized that the part discussing the state of Open Science in energy research aims to tackle a research question that requires a different approach from the one adopted in our paper. As such, answering this question outside the scope, so in order to avoid confusion, we remove the section comparing energy research with other disciplines. However, as it is an interesting research question worth mentioning, we have included it as further work in the Discussion section. 

**Issues** **Major Issues: ** 1. In the context of open source, the term and concept of research software is important and should be integrated (e.g. doi.org/10.3233/DS-1900262).    We thank the reviewer for the valuable reference. We have included the definition in the manuscript.  2. The third paragraph in section IV focuses on metadata. Here, a link to existing approaches for metadata in the energy domain would be valuable (e.g. doi.org/10.3390/en142066923).  Again, we thank the reviewer for the valuable reference. We have added information from the source in the manuscript.   3. The paper includes one figure. Unfortunately, the figure is not reproducible. The paper should reference to the actual dataset of the figure.    4. p6, "The number of entries for the energy category is remarkably low compared to other research fields": Well, it's a far more specialized field. This data is almost worthless without the number of publications or researchers in the respective fields for reference.   In response to the reviewers’ feedback in points 3 and 4, we have removed the figure and the analysis of the number of entries in the Horizon 2020 platform. As you correctly noted, a meaningful comparison would require a deeper analysis of the number of projects or researchers in each domain.  5. Publicly open conference are listed in the paragraph focusing on open educational resources. Scientific conferences are no educational resources. Also, it remains debatable if conference with conference fees can be counted as open.    As pointed out, scientific conferences are not educational resources. The new manuscript does not include them.   6. The citation style with hyperlinks and without further information makes the links unusable if printed. Also, some examples (e.g. OpenMod) are also missing links. The missing links should be added and all links should also marked in an additional way, e.g. footnotes.   We understand this issue. The original draft included the full URLs in footnotes. Unfortunately, the editing process modified them to include hyperlinks.  We will address this with the editors to consider the possibility of keeping the footnotes.  

**Minor Issues ** 1. Section III has a subsection A but no subsection B. We would recommend to remove the subheading from this subsection. Solved with the change in structure    2. The paper mainly uses the term energy research but sometimes also energy science. An uniform use of one term would improve the paper. The change was made in the text, in favour of energy research.   3. p4 "Allowing other researchers to reuse the data and methods [...] enhances the chance to detect bad research practices." -> Would the respective people use Open Science then? How does Open Science needs to be implemented to help with this problem?  This sentence is no longer part of the new manuscript. However, just to answer the questions, the idea of this argument is that reproducibility is encouraged. If so, “other researchers” can check whether the methods or the data correspond to the claims of the original authors.    4. p4/5, "barrier to implementing open access is related to the publishing industry revenue structure": Where exactly is the barrier here? The paragraph sounds like it's no problem at all.  We thank the reviewer for this comment. A similar point was brought forward by another reviewer. The analysis of the decoupling argument in the first version of the paper was misdirected. The paragraph has been re-written to better convey the original message put up by the referenced papers. We hope to have made the motivation and the argument clearer.  5. p6: Is Energies a high-profile journal? MDPI is considered to be predatory by many researchers. (e.g. https://academic.oup.com/rev/article/30/3/405/63481334)        This segment has been removed from the manuscript in the updated version.  6. p6, "Not pursuing openness...": This whole paragraph and the following one are difficult to understand.  This paragraph has been rewritten.
